# In silico insights into the adsorption and detection of dimethyltryptamine on metal doped C_20_ fullerene

**DOI:** 10.1038/s41598-026-60826-x

**Published:** 2026-07-22

**Authors:** Abdulrahman Sumayli, Saad M. Alshahrani, Jawaher Abdullah Alamoudi

**Affiliations:** 1 Department of Mechanical Engineering, College of Engineering, Najran University, Najran, Saudi Arabia; 2 Sustainability Lab, Scientific and Engineering Research Center (SERC), Najran University, Najran, Saudi Arabia; 3 Department of Pharmaceutical, College of Pharmacy, Princess Sattam Bin Abdulaziz University, P.O.Box 173, Al-Kharj, 11942 Saudi Arabia; 4 Department of Pharmaceutical Sciences, College of Pharmacy, Princess Nourah bint Abdulrahman University, P.O.Box 84428, Riyadh, 11671 Saudi Arabia

**Keywords:** Adsorption, Detection, C_20_ fullerene, Dimethyltryptamine, Sensor, DFT, Chemistry, Materials science

## Abstract

Given the psychoactive effects of dimethyltryptamine (DMT), classified as a Schedule I drug, developing diagnostic methods is essential. In this computational study, we investigate the adsorption and sensing behavior of DMT on pristine C_20_ fullerene and its beryllium (Be), magnesium (Mg), and calcium (Ca) doped derivatives using density functional theory (DFT), time-dependent DFT (TD-DFT), and quantum theory of atoms in molecules (QTAIM) to provide suitable candidates that reduce trial-and-error in subsequent experimental work. Three computational methods (B97D/lanl2dz, B97D/6-311 + G(d), and ωB97XD/lanl2dz) were employed to ensure the accuracy of qualitative results. Our results reveal that pristine C_20_ and BeC_19_ exhibit strong adsorption characterized by donor-acceptor interactions, electrostatic attraction, polarization/charge transfer, and dispersion forces. Electronic structure analysis indicates that BeC_19_ exhibits the largest reduction in Egap upon DMT adsorption, accompanied by pronounced charge transfer and substantial modulation of conductivity. UV-Vis spectra show that C_20_ and BeC_19_ exhibit a significant redshift from the UV to the visible region after DMT absorption, making them candidates for further experimental evaluation as colorimetric sensors. NCI, ELF, LOL, and QTAIM analyses confirm that interactions are predominantly non-covalent, with Be doping introducing localized attractive regions that enhance sensitivity. In contrast, MgC_19_ and CaC_19_ show minimal electronic perturbation and weak adsorption dominated by van der Waals forces. Statistical validation using MAD, RMSD, and Pearson correlation coefficients (*R* > 0.93) confirms good qualitative agreement across all computational methods. These theoretical findings suggest pure C_20_ as a potential adsorbent and BeC_19_ as a dual-purpose material for adsorption and electrochemical/colorimetric detection of DMT for further experimental work in the future, and provide a theoretical basis for the development of future experimental sensors.

**Supplementary Information:**

The online version contains supplementary material available at https://doi.org/10.1038/s41598-026-60826-x.

## Introduction

Dimethyltryptamine (DMT) is known worldwide for its remarkable psychedelic and hallucinogenic properties and has unique effects on human consciousness and perception. Although DMT is present in trace amounts in various plants and animal tissues, including the human brain, it is typically consumed to experience its psychoactive effects by smoking, orally ingesting edible forms, or via intravenous injection. Given its rapid onset and potent psychotropic effects, in many countries, DMT is classified as Schedule I, meaning it is not currently accepted for medical use and is a drug with high abuse potential^1,2^.

Recent analytical approaches have been reported for the accurate prediction of drug-target interactions^3^. Similarly, recent advances in analytical chemistry have introduced several innovative methods for detecting N, N‑dimethyltryptamine (DMT), reflecting a growing need for more sensitive, selective, and sustainable approaches in both clinical and forensic settings. For instance, Santos et al. developed a green dispersive liquid-liquid microextraction method for determining ayahuasca alkaloids, including DMT and β‑carbolines, in human hair, with a focus on reducing solvent consumption and environmental impact^4^. However, this technique is optimized for hair matrices, which limits its direct applicability to other biological specimens, such as blood or urine, without substantial methodological revalidation. In a different approach, Gomonit et al. employed LC-MS/MS to analyze DMT and its metabolites for forensic purposes, offering high sensitivity and specificity^5^. Nevertheless, the reliance on costly instrumentation and highly trained personnel restricts its routine use in resource‑limited forensic laboratories. Complementing these efforts, Motchaalangaram et al. combined electrogenerated chemiluminescence with a molecularly imprinted polymer to achieve sensitive and selective detection of DMT^6^. While this strategy enhances selectivity, the fabrication of molecularly imprinted polymers can be time‑consuming, and potential issues such as template leakage or batch‑to‑batch variability may compromise reproducibility. Collectively, these studies underscore significant progress in DMT detection but also highlight persistent challenges regarding matrix compatibility, cost, and manufacturing consistency that warrant further investigation.

Recent advances in nanostructured materials and drug-sensing platforms have underscored the importance of surface engineering and molecular-level interactions for improving detection sensitivity and selectivity. In particular, nanocarriers and functionalized surfaces have been extensively explored in pharmaceutical sciences for their tunable physicochemical properties, high surface area, and ability to form stable complexes with bioactive molecules, thereby enhancing both adsorption and detection performance. Studies have shown that interactions between small organic molecules and nanostructured systems are governed by electrostatic forces, hydrogen bonding, π-π interactions, and charge transfer processes, which collectively determine binding affinity and sensor response (e.g., in drug delivery and toxicological assessments)^7,8^. Moreover, recent developments in colloidal and surface chemistry emphasize that modifying nanomaterials through doping or surface functionalization can significantly alter their electronic structure, interfacial behavior, and adsorption capability, thereby improving their applicability in sensing and biomedical contexts^9^. In parallel, bioconjugation strategies have demonstrated that tailoring interactions between nanomaterials and biomolecules enhances selectivity and stability, which are critical for reliable detection systems^10^. These insights collectively support growing interest in designing advanced nanostructured platforms with optimized electronic and surface properties for sensitive detection of bioactive compounds.

There has been growing interest in recent years in developing electrochemical and colorimetric sensors for rapid, portable, on-site detection of psychoactive substances^11,12^. Carbon nanomaterials are notable for their high specific surface area, excellent electronic conductivity, and customizable chemical functionality. The fullerene family, particularly the prototypical C_60_ and the highly strained, geometrically distinct C_20_ buckyball, offers an exciting avenue for new sensor platforms. The C_20_ fullerene is especially noteworthy as the smallest stable fullerene with distinctive symmetry and electron affinity, making it suitable for sensing a number of molecular targets. Experimental work has also confirmed the synthesis of the C_20_ fullerene. Prinzbach et al. demonstrated early gas-phase production and photoelectron spectroscopy of C_20_, establishing its existence as the smallest member of the fullerene family^13^. More recently, Torrisi et al. reported the synthesis of fullerene C_20_ in carbon plasma produced by Nd: YAG laser ablation, further validating the feasibility of generating this elusive nanostructure under controlled laboratory conditions^14^. These confirmations open the door to exploring C_20_-based materials in next-generation sensing platforms.

Recently, the sensing and adsorption applications of C_20_ have been studied in various literature. For example, Rahimi et al. investigated the adsorption and sensing behavior of haloacetonitrile on the surface of C_20_ fullerene and highlighted the sensing capability of C_20_^15^. In another study, Alotaibi showed that doping C_20_ fullerenes with boron and nitrogen not only enhances their electronic properties but also makes them sensitive sensors for MDMA detection^16^. Similarly, Ghoreishi et al. investigated the sensing performance of pristine and nitrogen- and silicon-doped C_20_ nanocages for the detection of CO_2_, CO, N_2_, and H_2_ gases and highlighted not only the sensing capability of C20 but also the effect of doping in enhancing the sensing properties^17^. While these studies clearly demonstrate the potential of C_20_-based nanostructures for sensing various analytes, they primarily focus on small gas molecules or common drugs (e.g., MDMA, CO_2_, CO) and generally do not address complex indole-based psychoactive molecules such as DMT. Furthermore, most existing reports emphasize either adsorption energy or basic electronic descriptors, without providing a comprehensive multi-property sensing evaluation (electronic, optical, and conductivity-based) combined with cross-method validation. Therefore, there remains a gap in understanding how C_20_-based systems behave toward structurally complex psychoactive analytes and how dopant selection governs multi-modal sensing performance.

In this work, we investigate the interaction of pristine C_20_ and its doped forms with beryllium (Be), magnesium (Mg), and calcium (Ca) as hosts for DMT detection. The choice of these alkaline earth metals is justified by their distinct electronic and coordination properties. Beryllium (Be) is a chemical element in the periodic table that remains relatively underexplored, largely because of concerns about its reported toxicity and mutagenic potential. Despite these limitations, Be possesses several properties that make it highly relevant to coordination chemistry. Its small ionic radius, high charge density, and capacity to form stable complexes, including with biomolecules, suggest potential value in biorecognition applications. Compared with elements such as magnesium (Mg) and calcium (Ca), which can also coordinate with molecular species, Be often forms stronger interactions with specific ligands. This stronger binding behavior may improve selectivity toward target biomolecules, making Be an appealing candidate for biorecognition-based systems^18–21^. Magnesium doping is expected to induce semiconducting behavior in the C_20_ framework by altering the distributions of frontier molecular orbitals, potentially improving device selectivity and overall response rate while maintaining a moderate, reversible binding affinity. Calcium, due to its larger ionic radius and high polarizability, is also expected to facilitate efficient charge transfer between the adsorption site and the analyte, leading to strong interactions. Unlike beryllium, both magnesium and calcium exhibit lower toxicity, making them more suitable for designing biocompatible platforms, whereas studies have shown that beryllium doping is better suited to applications where selectivity is a priority^22^. Although in this work, Be, Mg, and Ca atoms were chosen as dopants, future work could investigate the effect of doping with other atoms. Expanding the spectrum of dopants would enable a more comprehensive understanding of the structure-property relationships in fullerene-based DMT sensors and could suggest superior candidates for future experimental validation.

To our knowledge, the ability of the C_20_ fullerene-based sensor to detect N, N‑dimethyltryptamine (DMT) has not yet been investigated in the experimental literature. Although several DFT-based studies have explored the adsorption and sensing behavior of small molecules on pristine and doped fullerene systems, the present work introduces several new aspects that distinguish it from existing literature. First, this study represents the first theoretical investigation of N, N-dimethyltryptamine (DMT) detection using C_20_-based fullerene systems, a target molecule that has not been previously examined in the context of nanostructured sensors. Second, unlike prior studies that typically focus on a single dopant or a limited set of dopants, this work provides a systematic comparative evaluation of alkaline-earth-metal dopants (Be, Mg, Ca), thereby enabling clear structure-property relationships that govern sensing performance. Third, the study integrates multi-level validation across three computational methods (B97D/lanl2dz, B97D/6–311 + G(d), and ωB97XD/lanl2dz), supported by statistical metrics (MAD, RMSD, and Pearson correlation), a practice rarely addressed in similar works, which helps ensure the robustness of the predicted trends. In addition, a comprehensive multi-technique analysis (DOS, TDOS/PDOS, NCI, ELF, LOL, QTAIM, UV-Vis, and conductivity) is employed to correlate adsorption strength with both electrochemical and colorimetric sensing responses. Finally, this work uniquely demonstrates that Be-doped C_20_ can act as a dual-function material (adsorbent+electrochemical/colorimetric sensor), whereas pristine C_20_ acts primarily as an adsorbent (providing a clear functional differentiation not previously reported). These combined aspects establish the present study as a methodologically robust and application-oriented extension of existing fullerene-based sensing research, specifically tailored toward DMT detection.

This computational work serves as a pre-synthesis step to reduce trial and error in future experimental efforts by identifying the most promising candidates for DMT sensing. It uses density functional theory (DFT), time-dependent density functional theory (TD-DFT), and quantum theory of atoms in molecules (QTAIM) to evaluate the electronic properties, excitation behavior, and intermolecular interactions between the DMT analyte and various C_20_-based hosts^23,24^.

To provide a deeper theoretical understanding of the adsorption mechanism, the interaction between DMT and metal-doped C_20_ can be rationalized in terms of four primary contributions: (i) donor–acceptor interactions, (ii) electrostatic attraction, (iii) polarization/charge transfer, and (iv) dispersion forces. The lone-pair electrons on the tertiary amine nitrogen of DMT act as a Lewis base, interacting with electron-deficient metal centers (particularly Be) through n→σ* donation from an empty orbital. This interaction is expected to be strongest for Be due to its small ionic radius and high charge density, which enhances orbital overlap. In contrast, Mg and Ca interactions are dominated by electrostatic and polarization effects due to their larger size and lower orbital overlap capability. For pristine C_20_, where no localized positive site exists, the interaction is governed primarily by dispersion forces and π-π type interactions between the fullerene cage and the indole moiety of DMT. These theoretical considerations provide a framework for interpreting the computed adsorption energies, charge transfer, and changes in electronic structure discussed in later sections. It should be noted that although dimethyltryptamine (DMT) is pharmacologically active, the purpose of this work is not to assess its pharmacological suitability or toxicological profile. Instead, this study focuses exclusively on the adsorption and sensing behavior of DMT using C_20_-based nanostructures. Advanced analyses such as ADMET prediction, Pred-hERG toxicity assessment, and pharmacokinetic modeling are beyond the scope of this work and may be considered in future studies to extend the applicability of these systems in pharmaceutical contexts^25^. We believe that the results of this theoretical work may serve as a valuable guide for future experimental work, potentially accelerating the development of portable, selective, and cost-effective DMT sensors for forensic or clinical applications.

## Computational section

In the first step, each of the structures in two states (1) isolated (C_20_, BeC_19_, MgC_19_, CaC_19_, and DMT (as analyte)) and (2) complex (C_20_@DMT, BeC_19_@DMT, MgC_19_@DMT, CaC_19_@DMT) was designed using GaussView 6.0 software, and the geometry of each was then optimized using Gaussian 09 W software^26^. All calculations were repeated using three computational methods: ωB97XD/lanl2dz, B97D/lanl2dz, and B97D/6–311 + G(d). The XYZ coordinates of the structures were reported in Table S1 of the Supplementary Data to ensure reproducibility of the results.

Solvent effects were modeled using the conductor-like polarizable continuum model (CPCM), a variant of the integral equation formalism polarizable continuum model (IEFPCM), as implemented in Gaussian 09 W. In this approach, the solute is placed in a cavity constructed from interlocking atomic spheres, and the surrounding aqueous phase (ε = 78.3553) is treated as a continuous dielectric medium. The CPCM solvation model was selected over other implicit solvation approaches (such as IEFPCM, SMD, or COSMO) for its improved numerical stability and efficiency in treating highly polar solvents like water. Compared to the traditional IEFPCM method, CPCM employs a conductor-like screening approximation that yields faster convergence and more stable electrostatic solutions, particularly for charged or highly polarizable systems. Additionally, CPCM has been widely validated for studying adsorption processes and electronic property modulation in nanostructured materials, making it a suitable choice for the present work. While more advanced solvation models such as SMD can incorporate additional non-electrostatic contributions (e.g., cavitation and dispersion), CPCM provides a reliable and computationally efficient framework for capturing the dominant electrostatic effects, which are the primary contributors in the systems investigated here. Therefore, CPCM represents an appropriate compromise between computational cost and accuracy for the qualitative objectives of this study. It is important to note that the CPCM implicit solvation model used in this study accounts only for the bulk electrostatic effects of the aqueous phase and does not explicitly describe specific solute–solvent interactions such as hydrogen bonding between solvent molecules and the DMT nitrogen sites. In an aqueous environment, DMT can form hydrogen bonds through its tertiary amine and indole nitrogen, which may compete with, or partially weaken, the interactions between DMT and the C_20_-based adsorbents. Consequently, the adsorption energies reported here may be slightly overestimated compared to a fully explicit aqueous environment. However, the implicit model still provides a reliable qualitative description of solvation effects on electronic properties, charge distribution, and relative interaction trends among the studied systems. Future studies incorporating explicit solvent molecules or hybrid QM/MM approaches would allow a more detailed evaluation of hydrogen-bonding networks and their influence on adsorption behavior in the aqueous phase^27,28^.

Additionally, the two density functionals, ωB97XD and B97D, were selected for their ability to account for noncovalent interactions. The choice of B97D and ωB97XD was further motivated by their proven reliability in modeling adsorption on carbon-based nanostructures and noncovalent host-guest systems, as reported in the literature. Long-range-corrected hybrid functionals such as ωB97XD are particularly important for accurately describing charge-transfer interactions and long-range electron-correlation effects, which are critical in adsorption and sensing mechanisms^29^. Meanwhile, generalized gradient approximation (GGA) functionals augmented with empirical dispersion corrections, such as B97D, provide computational efficiency while maintaining reasonable accuracy for large systems. The combined use of these functionals therefore ensures a balanced and validated description of both short-range and long-range interactions, reducing functional-dependent bias and increasing the robustness of the predicted trends^30^.

Repeating all calculations with different basis sets (lanl2dz and 6–311 + G(d)) and replicating the results across both functionals provides two major advantages. First, this approach assesses basis-set completeness and convergence, ensuring that the observed electronic properties and interaction energies are not artifacts of a particular basis-set choice. The lanl2dz basis set, which uses effective core potentials for heavier atoms, is computationally efficient and suitable for preliminary geometry optimization, whereas the larger 6–311 + G(d) basis set includes diffuse and polarization functions, providing higher accuracy for describing electron density in intermolecular regions^31^. Second, the qualitative agreement between the ωB97XD and B97D results strengthens confidence in the reported theoretical predictions and reduces the possibility of functional-specific bias. This cross-validation approach effectively identifies the most promising dopant candidates for subsequent experimental synthesis while minimizing false positives arising from methodological artifacts.

Finally, all geometric optimizations were performed without symmetry constraints and with the default convergence criteria in Gaussian 09. The optimization was considered converged when the maximum force, root-mean-square (RMS) force, maximum displacement, and RMS displacement fell below the standard thresholds (4.5 × 10^− 4^, 3.0 × 10^− 4^, 1.8 × 10^− 3^, and 1.2 × 10^− 3^ a.u., respectively). To ensure that all optimized structures correspond to true minima on the potential energy surface, vibrational frequency calculations were subsequently performed at the same level of theory for all isolated systems and complexes. The absence of imaginary frequencies in all cases confirms that the obtained geometries represent stable local minima rather than transition states.

The most important parameters for investigating structural and electronic properties, including energy gap (HLG), chemical softness (S), chemical hardness (η), chemical potential (µ), maximum charge transfer value (ΔNmax), and electrophilicity-based charge transfer (ECT), were calculated using Eqs. 1–6, respectively^32^.

1 $$\:\mathbf{H}\mathbf{L}\mathbf{G}=\left|{\mathbf{E}}_{\mathbf{H}\mathbf{O}\mathbf{M}\mathbf{O}}-{\mathbf{E}}_{\mathbf{L}\mathbf{U}\mathbf{M}\mathbf{O}}\right|$$

2 $$\:\boldsymbol{\upeta\:}=\raisebox{1ex}{$(-{\mathbf{E}}_{\mathbf{H}\mathbf{O}\mathbf{M}\mathbf{O}}-(-{\mathbf{E}}_{\mathbf{L}\mathbf{U}\mathbf{M}\mathbf{O}}\:\left)\right)$}\!\left/\:\!\raisebox{-1ex}{$2$}\right.$$

3 $$\:\boldsymbol{\upmu\:}=-(-{\mathbf{E}}_{\mathbf{H}\mathbf{O}\mathbf{M}\mathbf{O}}+(-{\mathbf{E}}_{\mathbf{L}\mathbf{U}\mathbf{M}\mathbf{O}}\left)\right)/2$$

4 $$\:\boldsymbol{S}=1/2\boldsymbol{\upeta\:}$$

5 $$\:{\varDelta\:\boldsymbol{N}}_{\boldsymbol{m}\boldsymbol{a}\boldsymbol{x}}=-\raisebox{1ex}{$\boldsymbol{\mu\:}$}\!\left/\:\!\raisebox{-1ex}{$\boldsymbol{\eta\:}$}\right.$$

6 $$\:\boldsymbol{E}\boldsymbol{C}\boldsymbol{T}={\left({\boldsymbol{\Delta\:}\boldsymbol{N}}_{\boldsymbol{m}\boldsymbol{a}\boldsymbol{x}}\right)}_{\boldsymbol{\alpha\:}}-{\left({\boldsymbol{\Delta\:}\boldsymbol{N}}_{\boldsymbol{m}\boldsymbol{a}\boldsymbol{x}}\right)}_{\boldsymbol{\beta\:}}$$

The energies of the highest occupied molecular orbital (HOMO) and the lowest unoccupied molecular orbital (LUMO) are represented by E_HOMO_ and E_LUMO_, respectively.

ΔNmax represents the maximum charge transfer, with α denoting the charge transfer for the complex and β denoting the charge transfer for the (Be/Mg/Ca)-doped C_20_. A positive ECT indicates that the sensor donates electrons to the DMT, whereas a negative ECT indicates that electrons transfer from the DMT to the sensor^33^.

The sensing properties were calculated and investigated using Eqs. 7–9.

7 $$\:{\mathbf{E}}_{\mathbf{a}\mathbf{d}\mathbf{s}}={\mathbf{E}}_{\left(\mathbf{R}-\right)\mathbf{C}20@\mathbf{D}\mathbf{M}\mathbf{T}}-\left({\mathbf{E}}_{\boldsymbol{D}\boldsymbol{M}\boldsymbol{T}}+{\mathbf{E}}_{\left(\mathbf{R}-\right)\mathbf{C}20}\right)+{\mathbf{E}}_{\mathbf{B}\mathbf{S}\mathbf{S}\mathbf{E}}$$

8 $$\:\boldsymbol{\tau\:}={\boldsymbol{V}}_{0}^{-1}\times\:\mathbf{e}\mathbf{x}\mathbf{p}(-\frac{{\boldsymbol{E}}_{\boldsymbol{a}\boldsymbol{d}\boldsymbol{s}}}{{\boldsymbol{k}}_{\boldsymbol{B}}\boldsymbol{T}})$$

9 $$\:\boldsymbol{\upsigma\:}=\boldsymbol{A}{\boldsymbol{T}}^{3/2}{\boldsymbol{e}}^{(-\boldsymbol{H}\boldsymbol{L}\boldsymbol{G}/2\boldsymbol{K}\boldsymbol{T})}$$

In Eq. (7), the adsorption energy (Eads) is computed from the total energy of the DMT-sensor complex (E_(R−)C20@DMT_), the energies of the isolated DMT (E_DMT_), and the sensor (E_(R−)C20_). The basis set superposition error (BSSE) was corrected using the Boys-Bernardi counterpoise method as implemented in Gaussian 09. The counterpoise correction was performed using the ‘counterpoise = 2’ keyword, treating the DMT molecule and the C_20_-based structures as two fragments. In this approach, each fragment’s energy was calculated in the full basis set of the complex, using ghost orbitals of the other fragment, and the corrected adsorption energies were obtained accordingly. Equation (8) defines the recovery time (τ) in terms of the adsorption energy, where V_0_ is the attempt frequency (typically ~ 10^12^ s^− 1^), kB is the Boltzmann constant, and T is the temperature (298 K). Lastly, Eq. (9) describes the electrical conductivity (σ), where A is the Richardson constant (6 × 10^5^ A.m^− 2^.K^− 2^), and T is the temperature (298 K)^34^.

Also, the dipole moment was calculated using Eq. 10.

10 $$\:\boldsymbol{\mu\:}=\sqrt{{\boldsymbol{\mu\:}}_{\boldsymbol{x}}^{2}+{\boldsymbol{\mu\:}}_{\boldsymbol{y}}^{2}+{\boldsymbol{\mu\:}}_{\boldsymbol{z}}^{2}}$$

µ is the total dipole moment magnitude, while µ_x_, µ_y_, and µ_z_ are its components along the x-, y-, and z-axes, respectively^35^.

We emphasize that the values calculated using the equations and theoretical methods in this work do not replace experimental results, as they are derived from computational models and inherent approximations. Therefore, these results are primarily intended for qualitative interpretation, trend identification, and comparative evaluation of different structures. Consequently, the final validation of the findings requires further experimental investigations. Nevertheless, we believe that theoretical approaches can significantly reduce the time and cost associated with experimental work by selecting the best candidates (by predicting key properties such as stability, reactivity, and optical behavior before synthesis), thereby minimizing unnecessary trial-and-error methods. Therefore, theoretical/computational methods can play a vital role in modern research. As such, we believe that the results of these computational studies effectively narrow the scope of potential candidates and provide reliable guidance for the design and conduct of future experimental research.

## Results and discussion

### Bond length and bond angle

**Fig. 1:**
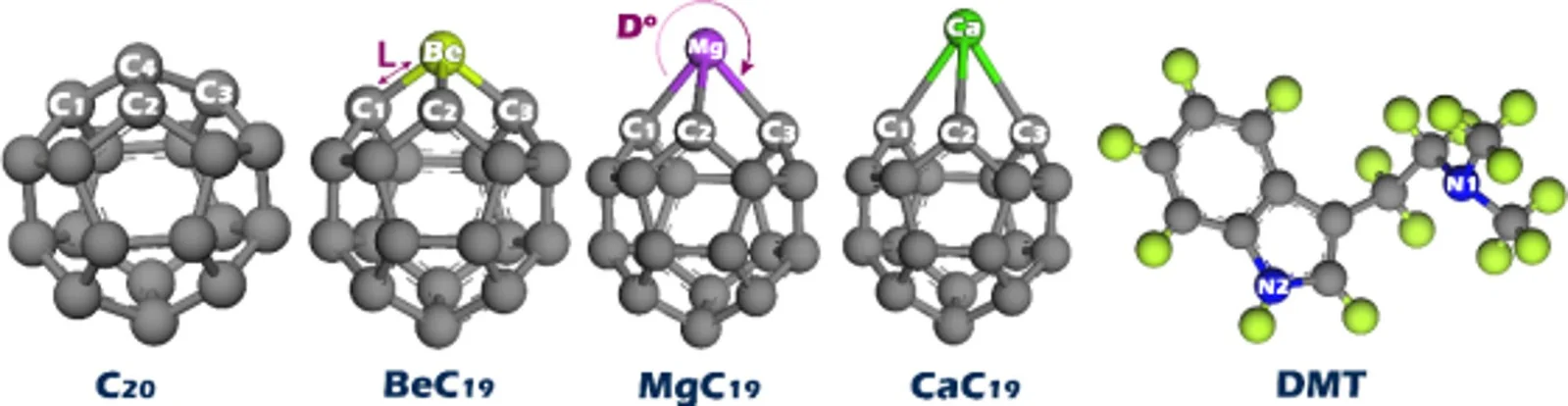
Optimized structure of each of the structures designed in this work.

The importance of structural parameters, such as specific bond lengths and bond angles, is recognized in defining both the three-dimensional shape and the chemical stability of molecular systems. The distance between two atoms plays a key role in determining bond strength: shorter bond lengths tend to be stronger. Bond angles determine the spatial arrangement and orientation of the bulk of the molecule, influencing both reactivity and physical properties. When a foreign atom is introduced into a carbon framework, such as C_20_, the redistribution of electrons and local deformations will significantly affect both bond lengths and bond angles^36^. In this regard, the bond lengths and bond angles of the initial and doped C_20_ structures were calculated before and after complex formation with DMT, and the results were reported in Fig. 1. and Table 1.

**Table 1:** Calculated bond length (Å) and bond angle (°) values for the initial C_20_ and MC_19_ metallofullerenes (M = Be, Ca, Mg) using three theoretical methods (B97D/lanl2dz, B97D/6–311 + G(d) and ωB97XD/lanl2dz).

B97D/lanl2dz				
Structure	**Bond length (Å)**		**Bond angle (** ^**o**^ **)**	
C_20_	C1-C4	1.471	C1-C4-C2	106.73
	C2-C4	1.533	C1-C4-C3	106.40
	C3-C4	1.429	C2-C4-C3	108.11
BeC_19_	Be-C1	1.805	C1-Be-C2	91.82
	Be-C2	1.806	C1- Be -C3	91.79
	Be-C3	1.807	C2- Be -C3	91.82
CaC_19_	Ca-C1	2.715	C1-Ca-C2	61.26
	Ca-C2	2.700	C1-Ca-C3	61.24
	Ca-C3	2.702	C2-Ca-C3	61.43
MgC_19_	Mg-C1	2.213	C1-Mg-C2	75.70
	Mg-C2	2.212	C1-Mg-C3	75.73
	Mg-C3	2.211	C2-Mg-C3	75.77

B97D/6–311 + G(d)				
Structure	Bond length (Å)		Bond angle (^o^)	
C_20_	C1-C4	1.417	C1-C4-C2	104.46
	C1-C3	1.437	C1-C4-C3	107.19
	C2-C3	1.534	C2-C4-C3	106.55
BeC_19_	Be-C1	1.797	C1-Be-C2	91.42
	Be-C2	1.799	C1- Be -C3	91.45
	Be-C3	1.798	C2- Be -C3	91.42
CaC_19_	Ca-C1	2.566	C1-Ca-C2	63.45
	Ca-C2	2.566	C1-Ca-C3	63.43
	Ca-C3	2.571	C2-Ca-C3	63.44
MgC_19_	Mg-C1	2.242	C1-Mg-C2	74.45
	Mg-C2	2.240	C1-Mg-C3	74.34
	Mg-C3	2.243	C2-Mg-C3	74.32

ωB97XD/lanl2dz				
Structure	Bond length (Å)		Bond angle (^o^)	
C_20_	C1-C4	1.413	C1-C4-C2	108.12
	C1-C3	1.526	C1-C4-C3	106.59
	C2-C3	1.460	C2-C4-C3	106.70
BeC_19_	Be-C1	1.791	C1-Be-C2	91.46
	Be-C2	1.791	C1- Be -C3	91.38
	Be-C3	1.792	C2- Be -C3	91.39
CaC_19_	Ca-C1	2.713	C1-Ca-C2	61.24
	Ca-C2	2.700	C1-Ca-C3	61.0
	Ca-C3	2.713	C2-Ca-C3	61.23
MgC_19_	Mg-C1	2.226	C1-Mg-C2	73.70
	Mg-C2	2.227	C1-Mg-C3	73.61
	Mg-C3	2.230	C2-Mg-C3	73.53

The analysis of the computational data in Table 1 reveals systematic structural changes in the MC_19_ (M = Be, Mg, Ca) metallofullerenes following substitutional doping of the C_20_ cage. From a scientific perspective, replacing a carbon vertex with a metal atom fundamentally alters the local bonding environment, strain distribution, and hybridization within the carbon framework. Doping has a stronger effect on the coordination region directly surrounding the heteroatom. In pristine C_20_, all carbon atoms are roughly equivalent, with strained three-coordinate environments due to the curvature and pentagonal ring junctions. This three-coordinate environment is fundamentally different from a tetrahedral (four-coordinate) geometry and should not be interpreted as tetrahedral despite similar bond angles. Upon substituting a carbon with Be, Mg, or Ca, the metal atom interacts with three adjacent carbon atoms of the cage, forming a threefold coordination environment. The observed bond lengths and angles indicate that the effect is most pronounced for Ca and Mg compared to Be, despite Be being smaller. For example, using the B97D/lanl2dz method, the Be-C bond lengths are approximately 1.806 Å, while Mg-C and Ca-C bonds are significantly longer at about 2.212 Å and 2.705 Å, respectively. This difference appears to arise because Ca and Mg interact primarily through electrostatic and polarization effects, causing further redistribution of electron density in adjacent C-C bonds. The C-C bonds opposite the dopant show marked elongation (up to 1.533 Å in Be-doped C20) compared to the shorter bonds adjacent to the dopant (1.429 Å), indicating that doping localizes strain: the metal atom relieves some angular tension but transfers it to specific C–C distances. The smaller Be atom is expected to exhibit stronger orbital overlap with the carbon cage, whereas larger atoms such as Ca tend to interact more through electrostatic and polarization effects. Consequently, doping affects coordination geometry and adjacent C-C bonds more than remote regions.

We also think that the observed structural changes are closely related to the effects of doping-induced rehybridization. In pristine C_20_, each carbon atom is three-coordinated and best described as predominantly sp²-hybridized but significantly pyramidalized due to the cage’s high curvature and intrinsic strain. The calculated bond angles (≈ 106–108°) deviate substantially from the ideal 120° expected for planar sp^2^ systems. Although these angles approach the tetrahedral value (109.5°), the C_20_ cage is not tetrahedral and does not exhibit sp3 hybridization. Instead, the observed angles arise from geometric constraints imposed by the highly strained pentagonal cage structure. Such deviations are a well-known feature of small fullerenes, in which curvature induces pyramidalization of sp^2^ carbon centers without yielding true tetrahedral geometry. Upon doping, the metal atom induces rehybridization of the three carbon atoms directly bonded to it. For Be-C_19_, the C-Be-C angles are about 91.8°, which is far from the tetrahedral 109.5° and even further from 90°, suggesting that the carbon atoms bearing the metal adopt a more p-rich hybrid (closer to sp^2^) to overlap with Be’s vacant orbitals. Meanwhile, the internal angles at carbon (C1-C4-C2 = 106.73°) remain nearly unchanged from pristine values, indicating that rehybridization is local. For Mg and Ca, the C-M-C angles are 75.7° and 61.3°, respectively (much smaller than for Be). Such acute angles imply an even greater s-character in the metal’s hybrid orbitals (or minimal covalent mixing), forcing the three surrounding carbons to tilt their σ-orbitals outward, thereby enhancing p-character in the C-M bonds. Consequently, these three carbons undergo partial rehybridization from near-sp3 toward sp^2^, as seen in the shortening of adjacent C = C-like distances (e.g., C3-C4 = 1.429 Å, which is shorter than a typical single bond). Thus, the dopant acts as a local hybridization modulator: smaller metals with covalent character (Be) preserve more sp^3^ character at carbon, while larger alkaline earth metals (Ca) induce strong ionic polarization and flattening of the carbon coordination sphere, increasing sp^2^ character.

Finally, although the numerical values differ among the three methods (B97D/lanl2dz, B97D/6–311 + G(d), and ωB97XD/lanl2dz), the qualitative trend remains the same across all calculations. Therefore, despite discrepancies arising from basis set completeness (lanl2dz vs. 6–311 + G(d)) and the fraction of exact exchange (B97D vs. ωB97XD), the physical interpretation (that doping induces localized rehybridization, alters bond strain asymmetrically, and exerts a greater effect on larger, more electropositive metals) is robust and method-independent. The overlap of the results from all three computational methods confirms the electronic effects due to doping, which could be beneficial for their absorption and sensing capabilities.

### MEP contours

The molecular electrostatic potential (MEP) contour provides important information about the points of the molecule that are more likely to participate in chemical interactions. Specifically, the red areas represent the most highly concentrated regions of electrons or negative electrostatic potential. These regions are typically near electronegative atoms and strongly favor electrophiles or hydrogen-bonding donors as potential substrates. On the opposite side of the spectrum, the blue regions depict low electron density or positive potential, which is generally located near hydrogen atoms or electropositive centers that can attract nucleophiles or electron-dense chemical moieties. The in-between area of green corresponds to neutral or weakly reactive regions of low chemical potential^37^. By evaluating these complementary color contours, it is possible to infer where chemical interactions could occur. Correspondingly, the MEP contour plots are provided in Fig. 3 for both the pristine C_20_ and its doped forms of Be, Mg, and Ca, and for DMT.

**Fig. 2:**
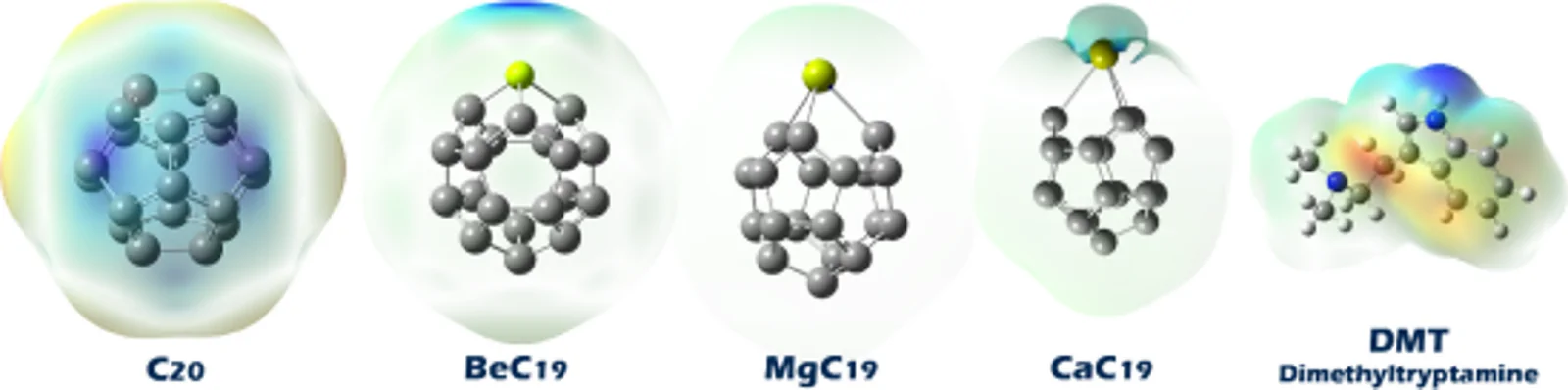
Molecular electrostatic potential (MEP) maps of (a) C_20_, (b) BeC_19_, (c) MgC_19_, (d) CaC_19_, and (e) DMT. Red indicates a negative potential (electron-rich) and blue indicates a positive potential (electron-deficient). For DMT, two nitrogen atoms are labeled: the tertiary amine (N_1_, adjacent to the two methyl groups) and the indole nitrogen (N_2_, part of the pyrrole ring).

The MEP surface of C_20_ shows a largely uniform electrostatic potential across the entire cage. The absence of strongly localized positive or negative regions indicates a delocalized electron density across the carbon framework. Consequently, no individual carbon atom is clearly identified as a preferential site based on electrostatic potential. In BeC_19_, the electrostatic potential map shows a well-defined region of positive potential localized around the Be atom. This feature clearly distinguishes the Be site from the surrounding carbon atoms and identifies it as the most electrostatically significant region of the structure. The MEP distribution of MgC_19_ reveals a positive electrostatic region centered on the Mg atom. This region is clearly discernible on the surface and marks Mg as the dominant site in terms of electrostatic potential within the cage. For CaC_19_, the MEP surface displays a pronounced positive region associated with the Ca atom. This region extends over a broader area surrounding the dopant, indicating that Ca constitutes the primary electrostatic feature of the structure (Fig. 2).

**Fig. 3:**
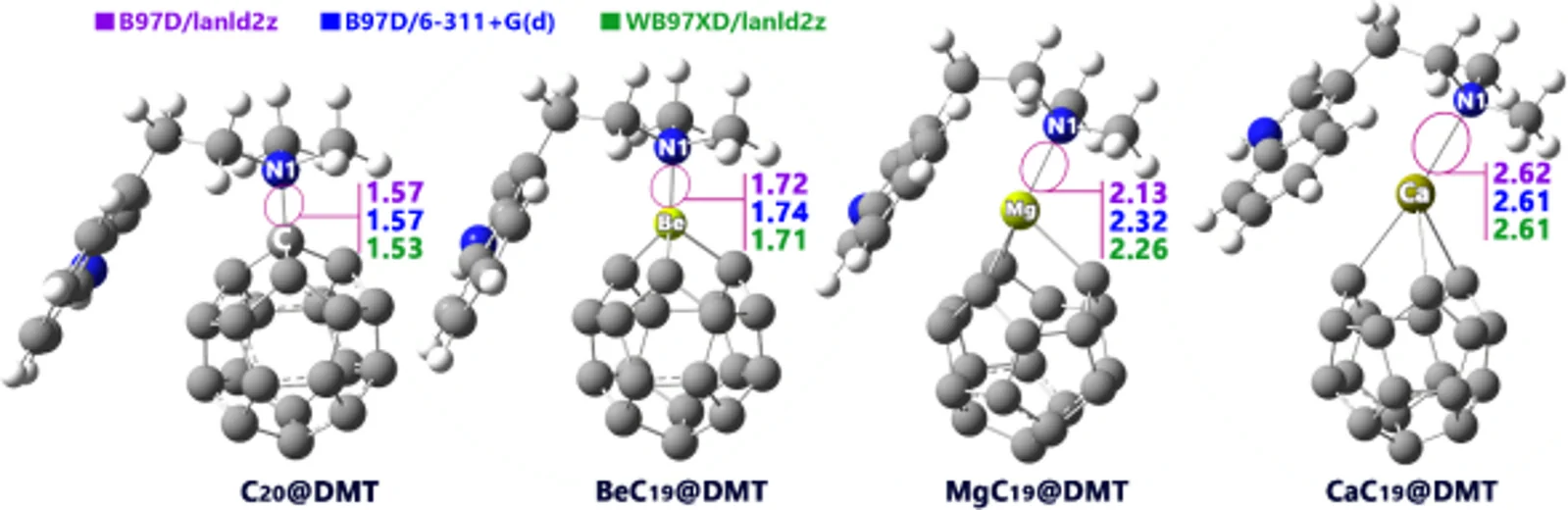
Optimized geometries of C_20_@DMT, BeC_19_@DMT, MgC_19_@DMT, and CaC_19_@DMT complexes showing the orientation of the dimethylamino nitrogen (N1) toward the cage surface or dopant atom. Selected N1···X (X = C, Be, Mg, Ca) distances (Å) are reported at the B97D/lanl2dz, B97D/6–311 + G(d), and ωB97XD/lanl2dz levels of theory.

N, N-Dimethyltryptamine (DMT) has two potential nitrogen coordination sites: the tertiary amine (N1, adjacent to the two methyl groups) and the indole nitrogen (N2, part of the pyrrole ring). For each system (C_20_, BeC_19_, CaC_19_, and MgC_19_), we constructed and optimized two conformers that differ in which nitrogen coordinates to the cage or metal-cage unit. Analysis of the adsorption energies (examined in detail in Sect. 3.3) showed that, in all cases, binding to the tertiary amine (N1) is more stable than binding to the indole N2. Accordingly, the more stable conformers (N1-bound) were retained for all subsequent analyses and discussion in the main text, while the less stable conformers (N2-bound) are presented in the Supplementary Data (Fig S1). The optimized geometries of the more stable complexes are shown in Fig. 3.

The optimized geometries indicate that the dimethylamino nitrogen atom (N1) of DMT is the primary contact site in all complexes. In C_20_@DMT, the short N1···C separation of 1.57 Å (B97D/lanl2dz), 1.57 Å (B97D/6–311 + G(d)), and 1.53 Å (ωB97XD/lanl2dz) suggests a strong attractive interaction between DMT and the pristine carbon cage, arising from close-range contact and additional dispersion contributions.

In BeC_19_@DMT, the N1 atom is oriented toward the Be dopant, with N1···Be distances of 1.72 Å (B97D/lanl2dz), 1.74 Å (B97D/6–311 + G(d)), and 1.71 Å (ωB97XD/lanl2dz). These distances indicate a strong, localized interaction, consistent with pronounced donor–acceptor character involving the nitrogen lone pair and the electron-deficient Be center.

For Mg-C_19_@DMT, the N1···Mg distances increase to 2.13 Å (B97D/lanl2dz), 2.32 Å (B97D/6–311 + G(d)), and 2.26 Å (ωB97XD/lanl2dz), indicating a weaker yet significant attractive interaction. The larger separation suggests reduced orbital overlap and a greater contribution from electrostatic and polarization effects.

In Ca-C_19_@DMT, the N1···Ca distances are 2.62 Å (B97D/lanl2dz), 2.61 Å (B97D/6–311 + G(d)), and 2.61 Å (ωB97XD/lanl2dz), the largest among the studied systems, indicating the weakest localized interaction. The attraction is more diffuse and dominated by long-range electrostatic contributions.

Across all systems, the three computational approaches show very close agreement in the optimized distances, indicating strong methodological consistency and reinforcing the reliability of the observed structural trends. Based on these results, the interaction strength is predicted to follow this trend: C_20_@DMT ≥ BeC_19_@DMT > MgC_19_@DMT > CaC_19_@DMT. Of course, this prediction requires more detailed studies, such as adsorption energy, which will be evaluated in more detail in the following sections.

### Reactivity parameters

Relevant descriptors of reactivity for chemical systems include the energy gap (Egap), chemical softness (S), hardness (η), chemical potential (µ), maximum charge transfer (ΔNmax), and electrophilicity-based charge transfer (ECT). The HLG provides valuable stability and reactivity indicators. The narrower the gap, the greater the reactivity; that is, softer species are more reactive, while harder species are less reactive and resist changes in the electron cloud. In the context of chemical sensing, a smaller Egap is directly associated with higher sensor sensitivity. This is because systems with a lower Egap require less energy for electronic excitation, enabling faster charge transfer upon analyte adsorption. As a result, even small interactions with target molecules can induce measurable changes in electrical conductivity or optical response, which is a key requirement for efficient sensor performance. Chemical potential indicates how easily an electron is added or removed from a molecule and how electrons will flow. The maximum charge transfer parameter quantifies how far electrons move in redox chemistry. Lastly, electrophilicity-based charge transfer quantifies the ability of molecules to accept electrons^38,39^. All of these parameters were determined for the systems discussed and have been summarized in Table 2.

**Table 2:** Calculated global reactivity descriptors for pristine and DMT-adsorbed C_20_ and M-C_19_ (M = Be, Mg, Ca) systems, including HOMO and LUMO energies (eV), energy gap (Egap, eV), chemical hardness (η, eV), chemical potential (µ, eV), chemical softness (S, eV^− 1^), maximum charge transfer (ΔNmax, dimensionless), and electrophilicity-based charge transfer (ECT, dimensionless).

B97D/Lanl2dz								
Structure	**HOMO**	**LUMO**	**Egap**	**η**	**µ**	**S**	**ΔNmax**	**ECT**
C_20_	−4.39	−5.03	0.64	0.32	−4.71	1.56	14.71	------
C_20_@DMT	−3.29	−3.99	0.70	0.35	−3.64	1.42	10.4	4.31
MgC_19_	−3.59	−5.28	1.69	0.84	−4.43	0.59	5.24	------
MgC_19_@DMT	−3.45	−5.02	1.57	0.78	−4.23	0.63	5.39	−0.14
CaC_19_	−3.18	−4.54	1.36	0.68	−3.86	0.73	5.67	------
CaC_19_@DMT	−3.15	−4.5	1.35	0.67	−3.82	0.74	5.66	0.009
BeC_19_	−4.04	−5.82	1.78	0.89	−4.93	0.56	5.53	------
BeC_19_@DMT	−3.54	−4.86	1.32	0.66	−4.2	0.75	6.36	−0.82

B97D/6–311 + G(d)								
Structure	HOMO	LUMO	Egap	η	µ	S	ΔNmax	ECT
C_20_	−3.93	−4.71	0.78	0.39	−4.32	1.28	11.07	------
C_20_@DMT	−3.09	−3.84	0.75	0.37	−3.46	1.33	9.24	1.83
MgC_19_	−3.26	−5.05	1.79	0.89	−4.15	0.55	4.64	------
MgC_19_@DMT	−3.16	−4.89	1.73	0.86	−4.02	0.57	4.65	−0.01
CaC_19_	−3.02	−4.68	1.66	0.83	−3.85	0.60	4.63	------
CaC_19_@DMT	−3.03	−4.69	1.66	0.83	−3.86	0.60	4.65	−0.01
BeC_19_	−3.66	−5.64	1.98	0.99	−4.65	0.50	4.69	------
BeC_19_@DMT	−3.27	−4.93	1.66	0.83	−4.1	0.60	4.93	−0.24

ωB97XD/Lanl2dz								
Structure	HOMO	LUMO	Egap	η	µ	S	ΔNmax	ECT
C_20_	−7.71	−2.48	5.23	2.61	−5.09	0.19	1.94	------
C_20_@DMT	−6.71	−1.33	5.38	2.69	−4.02	0.18	1.49	0.45
MgC_19_	−7.83	−1.58	6.25	3.12	−4.70	0.16	1.50	------
MgC_19_@DMT	−7.73	−1.47	6.26	3.13	−4.6	0.15	1.46	0.03
CaC_19_	−7.42	−1.18	6.24	3.12	−4.3	0.15	1.37	------
CaC_19_@DMT	−7.22	−1.04	6.18	3.09	−4.13	0.16	1.33	0.04
BeC_19_	−8.32	−2.1	6.22	3.11	−5.21	0.16	1.67	------
BeC_19_@DMT	−7.57	−1.66	5.91	2.95	−4.61	0.16	1.56	0.11

The electronic properties reported in Table 2 show noticeable variations upon DMT adsorption, allowing assessment of the sensitivity and adsorption capability of each structure. For C_20_, DMT adsorption induces significant changes in all electronic parameters. At the B97D/Lanl2dz level, the HOMO energy increases from − 4.39 to −3.29 eV and the LUMO from − 5.03 to −3.99 eV, accompanied by a slight increase in the energy gap (0.64 → 0.70 eV). The chemical potential (µ) becomes less negative (−4.71 → −3.64 eV), while softness (S) decreases (1.56 → 1.42) and ΔNmax drops considerably (14.71 → 10.4). A relatively large charge transfer (ECT = 4.31) is observed. Similar trends are maintained at the B97D/6–311 + G(d) and ωB97XD/Lanl2dz levels, confirming that C_20_ undergoes pronounced electronic perturbation upon DMT adsorption. For MgC_19_, the changes upon DMT adsorption are minimal. At B97D/Lanl2dz, Egap slightly decreases (1.69 → 1.57 eV), while µ and S show only marginal variations. The charge transfer is negligible (ECT = −0.14). This behavior is consistent across all computational levels, indicating that MgC_19_ is weakly sensitive to DMT. Similarly, CaC_19_ exhibits almost no variation in its electronic parameters upon adsorption. The HOMO, LUMO, Egap, η, µ, S, and ΔNmax values remain essentially unchanged at all levels of theory, with extremely small charge transfer (ECT ≈ 0). This suggests a very weak interaction and minimal electronic response. In contrast, BeC_19_ shows pronounced changes upon DMT adsorption. At B97D/Lanl2dz, Egap decreases significantly (1.78 → 1.32 eV), accompanied by a notable reduction in hardness (0.89 → 0.66) and an increase in softness (0.56 → 0.75). The chemical potential shifts markedly (−4.93 → −4.2 eV), and ΔNmax increases (5.53 → 6.36). A relatively large charge transfer (ECT = −0.82) is also observed. These trends are consistently reproduced at the other computational levels, confirming substantial electronic reorganization upon adsorption. From a theoretical perspective, the observed changes in Egap, µ, and ΔNmax indicate the extent of electronic coupling and charge transfer between DMT and the adsorbent. In this regard, the significant changes observed (especially Egap) for BeC_19_ and C_20_ upon DMT adsorption indicate increased electronic sensitivity of the system. From a sensing perspective, this narrowing of the band gap facilitates charge transfer between the analyte and the sensor surface, leading to more pronounced changes in measurable properties such as conductivity. Thus, a smaller band gap predicts the potential of BeC_19_ and C_20_ as electrochemical sensors for DMT detection. In contrast, based on the minimal changes observed for MgC_19_ and CaC_19_, their interactions are expected to be more influenced by electrostatic contributions and weaker dispersion than by significant orbital overlap.

**Fig. 4:**
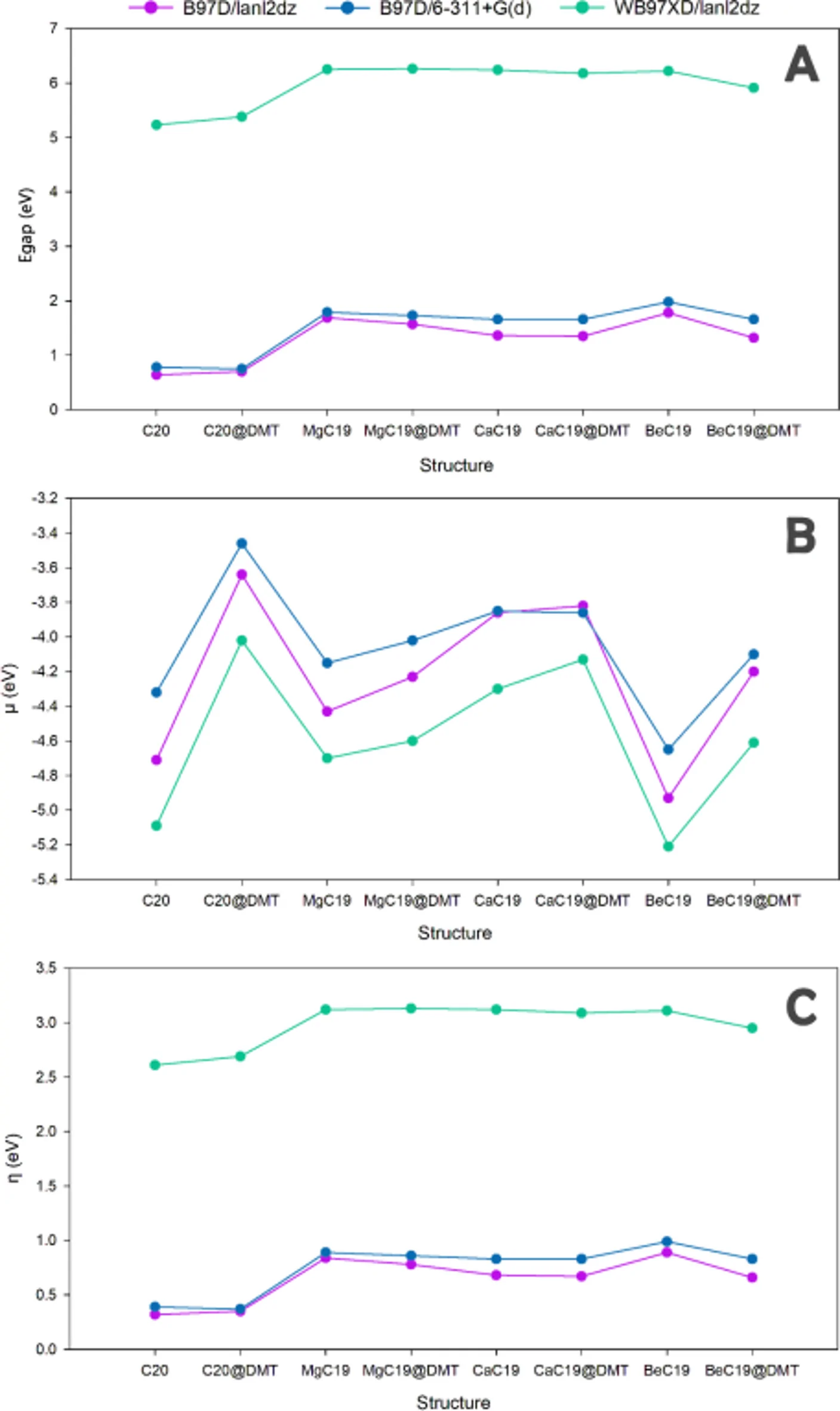
Variation of (A) Energy gap (Egap), (B) chemical potential (µ), and (C) chemical hardness (η) for pristine and DMT-adsorbed C_20_, BeC_19_, MgC_19_, and CaC_19_ structures, calculated at B97D/lanl2dz, B97D/6–311 + G(d), and ωB97XD/lanl2dz levels of theory.

Figure 4 (A-C) shows changes in key descriptors of global reactivity (Egap, chemical potential (µ), and chemical hardness (η)) for the pristine and DMT-adsorbed systems, calculated at three levels of theory. A consistent qualitative agreement is observed across all methods, confirming the robustness of the trends.

In Fig. 4-A (Egap), the pristine C_20_ system exhibits the smallest energy gap, which changes only slightly upon DMT adsorption. In contrast, the doped systems (MgC_19_, CaC_19_, and BeC_19_) show significantly larger gaps. Upon adsorption, only minor variations are observed for MgC_19_ and CaC_19_, whereas BeC_19_ shows a noticeable decrease in HLG, indicating a stronger electronic response to DMT. Across all systems, the ωB97XD/lanl2dz method systematically predicts larger gaps due to its treatment of exchange, while preserving the same trend.

In Fig. 4-B (chemical potential, µ), DMT adsorption generally shifts µ toward less negative values, particularly for C_20_ and BeC_19_, indicating a redistribution of electron density upon interaction. The change is minimal for MgC_19_ and CaC_19_, further suggesting a weaker interaction with DMT. Despite differences in absolute values, all three computational methods reproduce the same pattern of variation.

In Fig. 4-C (chemical hardness, η), a clear reduction in hardness is observed for BeC_19_ upon adsorption, whereas MgC_19_ and CaC_19_ show only marginal changes. Because lower hardness is associated with higher reactivity, this result highlights the enhanced sensitivity of BeC_19_ to DMT. The pristine C_20_ system remains relatively soft, with only slight variation after adsorption.

Overall, the combined analysis of Egap, µ, and η consistently indicates that BeC_19_ undergoes the most significant electronic perturbation upon DMT adsorption, followed by C_20_, whereas MgC_19_ and CaC_19_ show minimal changes. The agreement among all three computational approaches reinforces the conclusion that BeC_19_ and C_20_ may be promising candidates for DMT absorption and sensing applications.

**Fig. 5:**
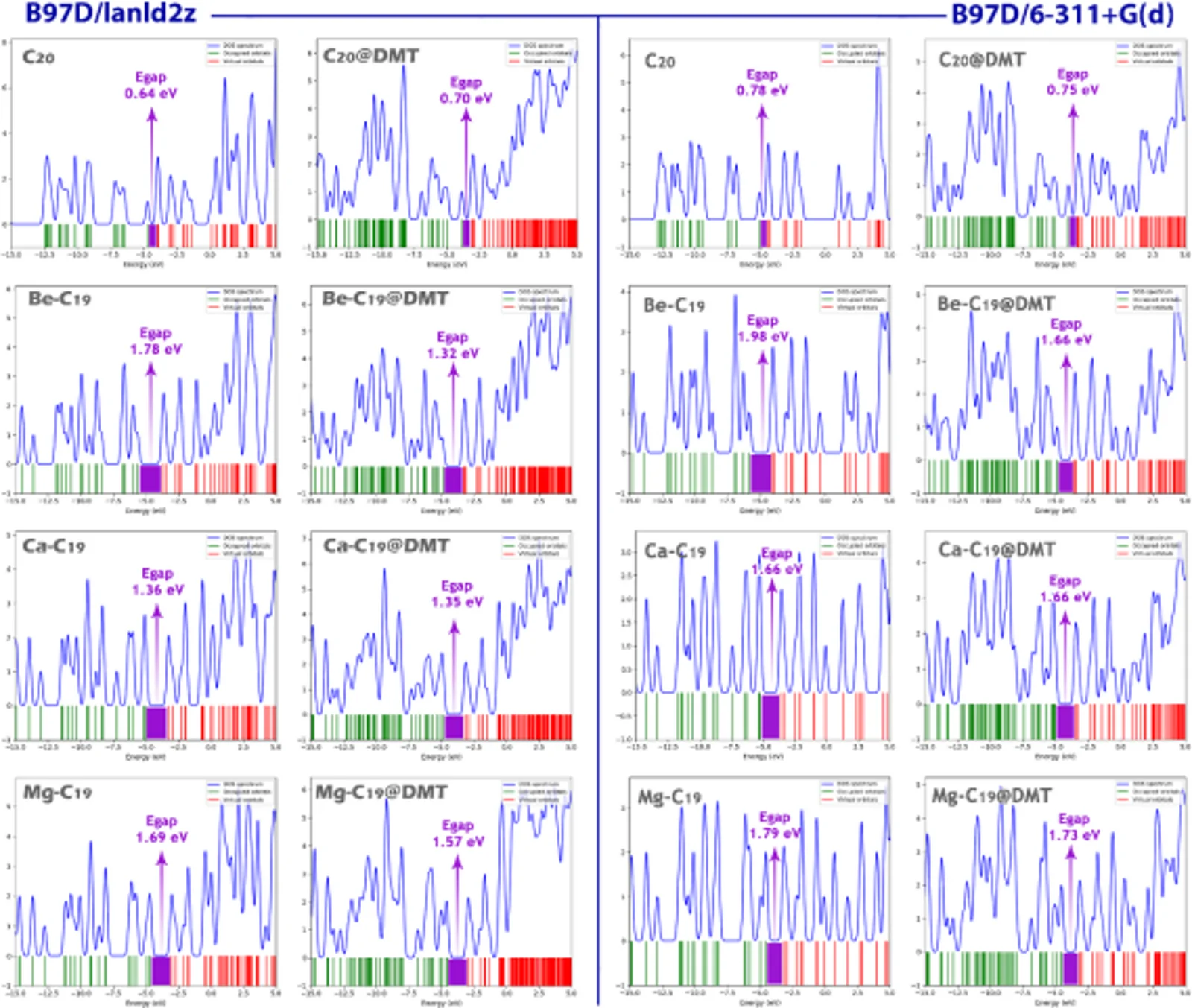
Density of states (DOS) plots of C_20_, BeC_19_, MgC_19_, and CaC_19_ before and after DMT adsorption, calculated at the B97D/lanl2dz and B97D/6–311 + G(d) levels of theory, showing changes in the electronic structure and Egap upon interaction with the analyte.

Analyzing density of states (DOS) plots is essential for understanding the electronic structure of materials, particularly the distribution of occupied and unoccupied energy levels and the nature of the Egap. DOS plots provide a visual representation of how electronic states are populated around the Fermi level and allow direct assessment of changes in electronic behavior upon adsorption, such as gap modulation, orbital redistribution, and the emergence of new states. These features are directly related to key reactivity descriptors such as Egap, chemical potential, and charge transfer (Fig. 5)^40^.

Inspection of the DOS plots shows that, for C_20_, DMT adsorption induces a slight shift in the frontier orbital region and a small change in the gap (0.64 → 0.70 eV at B97D/lanl2dz and 0.78 → 0.75 eV at B97D/6–311 + G(d)). The overall DOS profile is perturbed, with a redistribution of states near the Fermi level. This behavior is consistent with Table 2, which reports moderate changes in HOMO/LUMO energies and chemical potential, along with noticeable charge transfer, confirming a measurable electronic response.

For BeC_19_, the DOS plots show a clear reduction in the energy gap upon DMT adsorption (1.78 → 1.32 eV and 1.98 → 1.66 eV). In addition, new states appear near the Fermi level, indicating significant orbital mixing between the cage and the analyte. This pronounced change in the DOS is in excellent agreement with Table 2, which reports a substantial decrease in Egap, reduced hardness, increased softness, and significant charge transfer. Together, these features confirm strong electronic interaction and high sensitivity.

In contrast, the DOS plots of MgC19 show only minor changes upon adsorption, with a small decrease in the gap (1.69 → 1.57 eV and 1.79 → 1.73 eV). The state distribution remains largely unchanged, and no significant new features appear near the Fermi level. This observation aligns with the tabulated data, which show negligible variations in electronic descriptors and minimal charge transfer.

Similarly, for CaC_19_, the DOS spectra before and after adsorption are nearly identical, and the energy gap remains essentially unchanged (1.36 → 1.35 eV and 1.66 → 1.66 eV). The absence of noticeable changes in the DOS confirms the very weak electronic perturbation reported in Table 2, including nearly unchanged HOMO/LUMO energies and negligible charge transfer.

A consistent qualitative trend is observed across the two computational approaches using the same functional (B97D) with different basis sets (Lanl2dz and 6–311 + G(d)), as both reproduce the same pattern of DOS modifications and gap variations upon adsorption. In addition, DOS spectra were calculated using the ωB97XD/lanl2dz method. Although this approach yields different absolute energy values, it reproduces the same qualitative trends observed in the B97D-based calculations, confirming the robustness of the results. The larger energy gaps predicted by ωB97XD arise from its inclusion of long-range exact (Hartree-Fock) exchange, which reduces self-interaction errors and improves the separation between HOMO and LUMO energies. In contrast, B97D lacks exact exchange and tends to underestimate the gap due to increased electron delocalization. The DOS spectra calculated with the ωB97XD/lanl2dz method are presented in the Supplementary Data (Fig. S2).

Overall, the DOS analysis fully supports the trends observed in Table 2. The most significant changes in electronic structure occur for BeC19, followed by moderate changes for C_20_, while MgC_19_ and CaC_19_ remain largely unaffected. This consistency between DOS characteristics and global reactivity descriptors reinforces the conclusion that BeC_19_ and C_20_ may perform better in DMT adsorption.

**Fig. 6:**
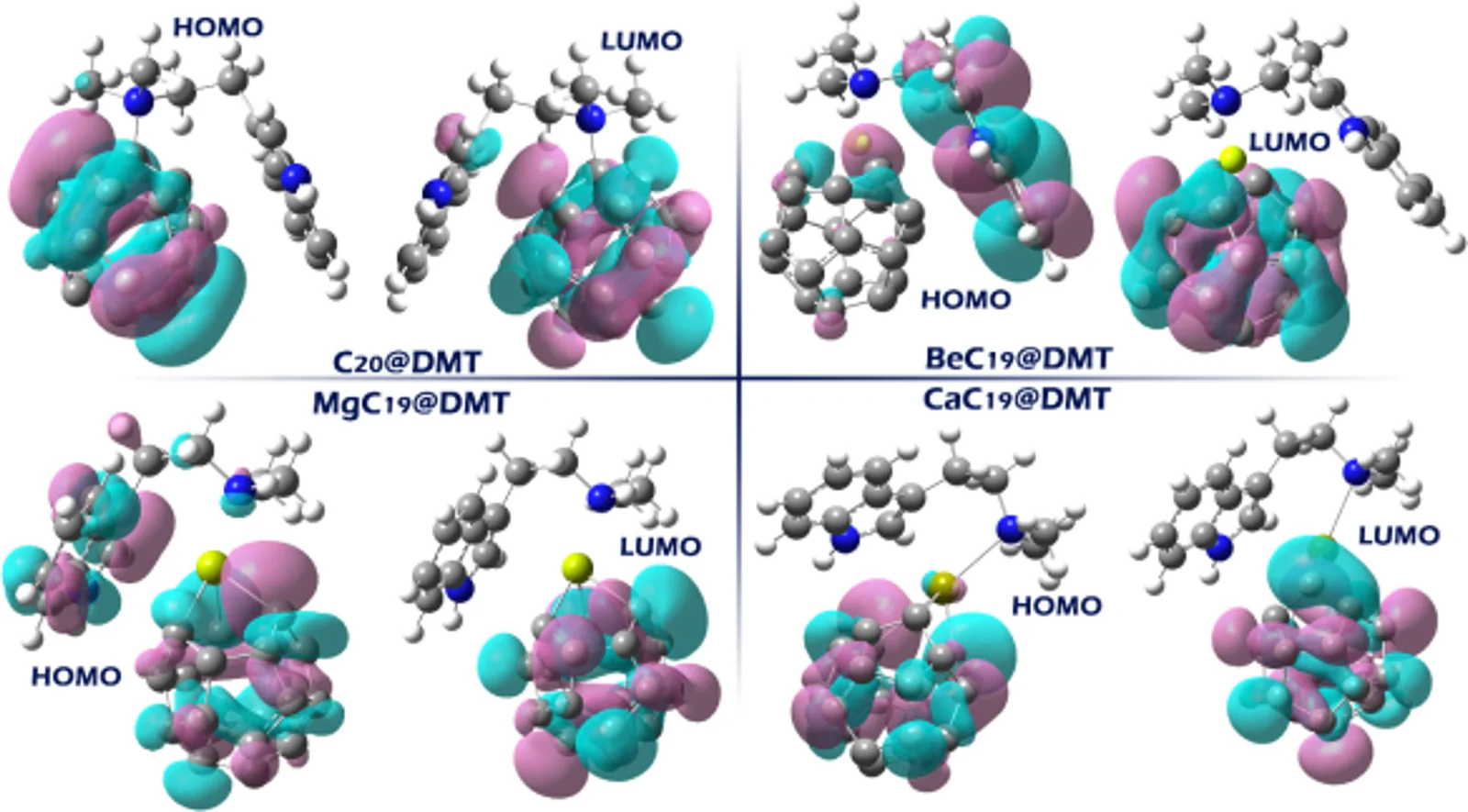
Frontier molecular orbital (HOMO and LUMO) distributions of C_20_@DMT, BeC_19_@DMT, MgC_19_@DMT, and CaC19@DMT complexes, illustrating the spatial localization of electronic density. The cyan and magenta isosurfaces represent the positive and negative phases of the molecular orbitals (calculated using the B97D/lanl2dz method).

Analyzing the spatial distribution of the HOMO and LUMO orbitals provides valuable information about the specific sites and pathways of electronic interactions, including charge-transfer mechanisms during sensing processes (Fig. 6).

In C_20_@DMT, the HOMO is predominantly localized on the carbon cage, with only a minor contribution from the DMT molecule. In contrast, the LUMO is distributed between the cage and the DMT moiety, indicating partial orbital delocalization at the interface. This distribution suggests that the cage plays a dominant role in the electron-donating character, while the accepting orbital begins to involve both fragments.

In BeC19@DMT, a clear redistribution of the frontier orbitals is observed. The HOMO is primarily localized on the DMT molecule, particularly around the nitrogen-containing region, whereas the LUMO is concentrated on the Be-doped cage. The spatial separation of HOMO (localized on DMT) and LUMO (localized on BeC_19_) in the Be-doped system is a clear signature of a donor-acceptor interaction mechanism, in which electron density can be transferred from the nitrogen lone pair into the acceptor states of the doped cage. This orbital alignment facilitates charge transfer and predicts increased sensitivity and absorption power for BeC_19_.

In MgC_19_@DMT, the HOMO remains largely localized on the cage, with only a minor contribution from DMT. The LUMO is also primarily centered on the cage, especially near the Mg dopant, with minimal extension toward the analyte. This indicates weak orbital overlap between the two components and limited electronic communication.

A similar behavior is observed for CaC_19_@DMT, where both HOMO and LUMO are mainly localized on the Ca-doped cage. The DMT molecule contributes very little to the frontier orbitals, reflecting minimal interaction and weak electronic coupling between the two fragments.

Overall, the degree of HOMO-LUMO separation and delocalization follow the trend: Be–C_19_@DMT > C_20_@DMT > MgC_19_@DMT ≈ CaC_19_@DMT. The pronounced spatial separation in BeC_19_@DMT indicates the strongest electronic interaction, whereas the strong localization on the cage in MgC_19_@DMT and CaC_19_@DMT confirms their weak interaction with DMT.

Analysis of total density of states (TDOS) and predicted density of states (PDOS) contours is fundamental to elucidating the electronic structure and interaction mechanism in adsorbent–analyte systems. TDOS provides the overall distribution of electronic states, particularly near the Fermi level, which governs reactivity and sensitivity. In contrast, OPDOS directly reflects the nature of orbital interactions between fragments: positive values indicate bonding interactions, while negative values correspond to antibonding contributions. Decomposition into s and p orbitals further clarifies the origin of these interactions: p orbitals typically dominate directional bonding and frontier orbital behavior, whereas s orbitals contribute weakly and isotopically. Such analysis is therefore crucial for rational design, as strong adsorption is generally associated with significant TDOS redistribution near the Fermi level and pronounced OPDOS features^41^.

**Fig. 7:**
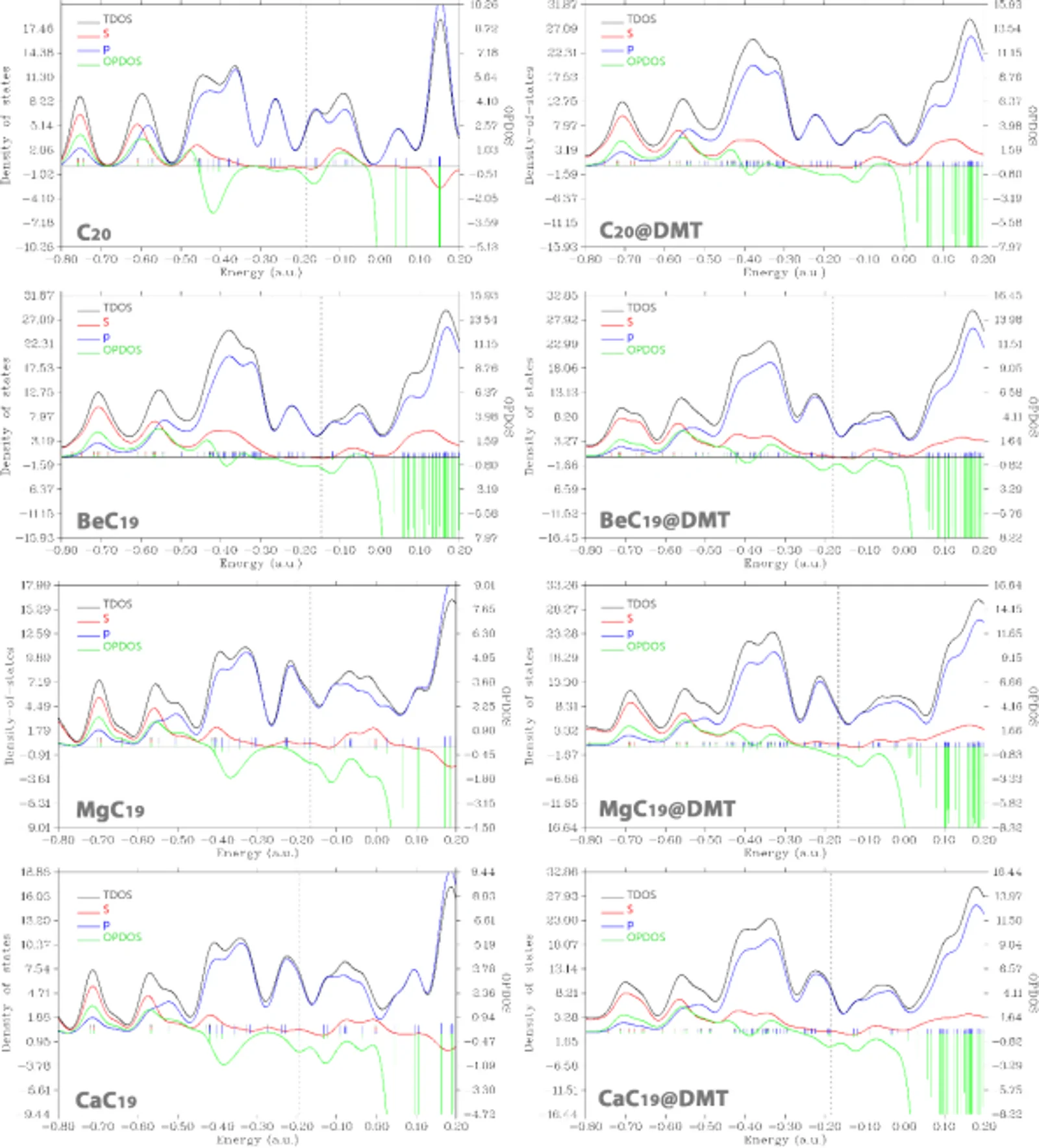
Total density of states (TDOS), projected density of states (s and p contributions), and overlap population density of states (OPDOS) for C_20_, BeC_19_, MgC_19_, and CaC_19_ before and after DMT adsorption, calculated at the B97D/lanl2dz level of theory, illustrating orbital contributions and interaction characteristics near the Fermi level.

According to Fig. 7 (and the spectra calculated with the ωB97XD/lanl2dz and B97D/6–311 + G(d) methods presented in Fig. S3 and Fig. S4 in the Supplementary Data), all complexes show that the p-orbitals dominate the electronic structure, as the p-DOS closely follows the TDOS across the energy range, especially near the Fermi level. The s-orbital contribution is relatively insignificant, indicating that the interaction with DMT is primarily governed by the p-type electronic states of the cages and the analyte. For C_20_, DMT adsorption causes a clear redistribution of the TDOS near the Fermi surface, accompanied by significant OPDOS features with both bonding and antibonding character, indicating substantial orbital overlap between DMT and the carbon cage. In BeC_19_, the most pronounced changes are observed. The TDOS shows significant reorganization near the Fermi level after adsorption, and the OPDOS exhibits strong positive and negative peaks, indicating substantial orbital overlap and interaction. This confirms that Be doping enhances electronic coupling with DMT, consistent with strong adsorption behavior. For MgC_19_, the TDOS profiles before and after adsorption remain largely similar, with only slight changes near the Fermi level. The OPDOS features are weak, indicating limited orbital interaction and weak adsorption. Similarly, CaC_19_ shows minimal differences in TDOS upon adsorption, and the OPDOS remains weak and largely unchanged, confirming negligible interaction with DMT.

Importantly, all three computational methods show strong qualitative agreement. Both B97D/lanl2dz and B97D/6–311 + G(d) reproduce the same trends in TDOS redistribution and OPDOS intensity, and the ωB97XD/lanl2dz results, despite differences in absolute energy scales, show the same qualitative behavior. This consistency demonstrates that the observed interaction trends are method-independent. Overall, the interaction strength inferred from TDOS/OPDOS analysis follows the order: BeC_19_ > C_20_ > MgC_19_ ≈ CaC_19_, which is fully consistent with the previously discussed electronic descriptors.

### Adsorption energy, Recovery time, and Electrical conductivity

Sensor performance is primarily governed by adsorption energy, recovery time, and electrical conductivity. Adsorption energy (Eads) quantifies the interaction strength between the sensor and the target molecule, with optimal intermediate values ensuring stable adsorption and easy desorption for cyclic sensing. Recovery time, which depends on adsorption strength, reflects how quickly the sensor returns to its initial state after analyte detection, thereby affecting reusability and efficiency. Electrical conductivity indicates the sensor’s ability to convert molecular interactions into measurable electrical responses, determining sensitivity and responsiveness^42^. Computational analysis of these parameters for the designed sensors and their DMT complexes is summarized in Table 3 (the reported values correspond to the nanocage orientation toward N1 in DMT). The adsorption energy values for each structure attached to the indole nitrogen (N2) are reported in Table S2 of the Supplementary Data.

**Table 3:** Adsorption energies (Eads), basis set superposition error (BSSE), recovery times (τ), and electrical conductivities (σ) for pristine and DMT-adsorbed C20, BeC19, MgC19, and CaC19 structures, computed at the B97D/lanl2dz, B97D/6–311 + G(d), and ωB97XD/lanl2dz levels of theory.

B97D/lanl2dz						
Structure	**Eads (kcal.mol** ^**− 1**^ **)**	**BSSE** **(kcal.mol** ^**− 1**^ **)**	**τ (s)**	**Log**_**10**_ **(τ)**	**σ (Sm** ^**− 1**^ **)**	**Log**_**10**_ **(σ)**
C_20_	**--------**	**--------**	**--------**	**--------**	1.2 × 10^4^	4.07
MgC_19_	**--------**	**--------**	**--------**	**--------**	1.58 × 10^− 5^	−4.79
CaC_19_	**--------**	**--------**	**--------**	**--------**	9.78 × 10^− 3^	−2.01
BeC_19_	**--------**	**--------**	**--------**	**--------**	2.75 × 10^− 6^	−5.56
C_20_@DMT	−53.58	4.12	2.04 × 10^27^	27.31	3.72 × 10^3^	3.57
MgC_19_@DMT	−28.43	4.92	7.24 × 10^8^	8.86	1.64 × 10^− 4^	−3.78
CaC_19_@DMT	−13.16	3.16	4.57 × 10^− 3^	−2.34	1.19 × 10^− 2^	−1.92
BeC_19_@DMT	−44.54	7.39	4.79 × 10^20^	20.68	2.13 × 10^− 2^	−1.67
B97D/6–311 + G(d)						
C_20_	**--------**	**--------**	**--------**	**--------**	7.84 × 10^2^	2.89
MgC_19_	**--------**	**--------**	**--------**	**--------**	2.27 × 10^− 6^	−5.64
CaC_19_	**--------**	**--------**	**--------**	**--------**	2.85 × 10^− 5^	−4.54
BeC_19_	**--------**	**--------**	**--------**	**--------**	5.60 × 10^− 8^	−7.25
C_20_@DMT	−42.54	1.25	1.62 × 10^19^	19.21	1.41 × 10^3^	3.14
MgC_19_@DMT	−26.34	3.51	2.14 × 10^7^	7.33	7.28 × 10^− 6^	−5.13
CaC_19_@DMT	−11.12	1.88	1.45 × 10^− 4^	−3.84	2.85 × 10^− 5^	−4.54
BeC_19_@DMT	−42.37	3.13	1.20 × 10^19^	19.08	2.85 × 10^− 5^	−4.54
ωB97XD/lanl2dz						
C_20_	**--------**	**--------**	**--------**	**--------**	1.86 × 10^− 35^	−34.73
MgC_19_	--------	**--------**	**--------**	**--------**	4.43 × 10^− 44^	−43.35
CaC_19_	--------	**--------**	**--------**	**--------**	5.28 × 10^− 44^	−43.27
BeC_19_	--------	**--------**	**--------**	**--------**	7.93 × 10^− 44^	−43.10
C_20_@DMT	−53.40	9.96	1.48 × 10^27^	27.17	1.00 × 10^− 36^	−35.99
MgC_19_@DMT	−27.19	4.45	8.71 × 10^7^	7.94	3.64 × 10^− 44^	−43.43
CaC_19_@DMT	−12.93	3.83	3.02 × 10^− 3^	−2.52	1.37 × 10^− 43^	−43.27
BeC_19_@DMT	−31.30	21.73	9.12 × 10^10^	10.96	3.31 × 10^− 41^	−40.48

Adsorption strength shows a clear trend across all computational methods. At the B97D/lanl2dz level, C_20_@DMT (−53.58 kcal.mol^− 1^) and BeC19@DMT (−44.54 kcal.mol^− 1^) have significantly more negative adsorption energies than MgC_19_@DMT (−28.43 kcal.mol^− 1^) and CaC_19_@DMT (−13.16 kcal.mol^− 1^). This trend persists at B97D/6–311 + G(d) [C_20_@DMT (−42.54), BeC_19_@DMT (−42.37), MgC_19_@DMT (−26.34), CaC_19_@DMT (−11.12) kcal.mol^− 1^] and ωB97XD/lanl2dz [C_20_@DMT (−53.40), BeC_19_@DMT (−31.30), MgC_19_@DMT (−27.19), CaC_19_@DMT (−12.93) kcal.mol^− 1^]. The consistently large negative values for C_20_ and BeC_19_ indicate strong adsorption, whereas MgC_19_ and especially CaC_19_ show weaker binding. Thus, both C_20_ and BeC_19_ are potential adsorbents for DMT removal (Table 3).

Changes in conductivity after adsorption were investigated as a key indicator for sensing applications. For C_20_, conductivity decreases significantly upon adsorption: from 1.2 × 10^4^ Sm^− 1^ (B97D/lanl2dz) and 7.84 × 10^2^ Sm^− 1^ (B97D/6–311 + G(d)) to 3.72 × 10^3^ Sm^− 1^ and 1.41 × 10^3^ Sm-1, respectively. A similar trend is observed at ωB97XD/lanl2dz (1.86 × 10–35 → 1.00 × 10^− 36^ Sm^− 1^). For BeC19, a dramatic increase in conductivity is observed upon adsorption: from 2.75 × 10^− 6^ Sm^− 1^ (B97D/lanl2dz) and 5.60 × 10^− 8^ Sm^− 1^ (B97D/6–311 + G(d)) to 2.13 × 10^− 2^ Sm^− 1^ and 2.85 × 10 − 5 Sm^− 1^, respectively; similarly, at ωB97XD/lanl2dz (7.93 × 10^− 44^ → 3.31 × 10^− 41^ Sm^− 1^). This substantial modulation of conductivity indicates strong sensitivity. In contrast, MgC^19^ and CaC19 exhibit only minor changes in conductivity across all methods, confirming their weak response to DMT.

At the B97D/lanl2dz level, extremely long recovery times are observed for C20@DMT (2.04 × 10^27^ s) and BeC_19_@DMT (4.79 × 10^20^ s), indicating very slow desorption. Similar behavior is observed at B97D/6–311 + G(d) [C_20_@DMT (1.62 × 10^19^ s), BeC_19_@DMT (1.20 × 10^19^ s)] and ωB97XD/lanl2dz [C_20_@DMT (1.48 × 10^27^ s), BeC_19_@DMT (9.12 × 10^10^ s)]. In contrast, MgC_19_@DMT and CaC_19_@DMT exhibit much shorter recovery times (e.g., 7.24 × 10^8^ and 4.57 × 10^− 3^ s at B97D/lanl2dz), suggesting easier desorption but weaker adsorption.

**Fig. 8:**
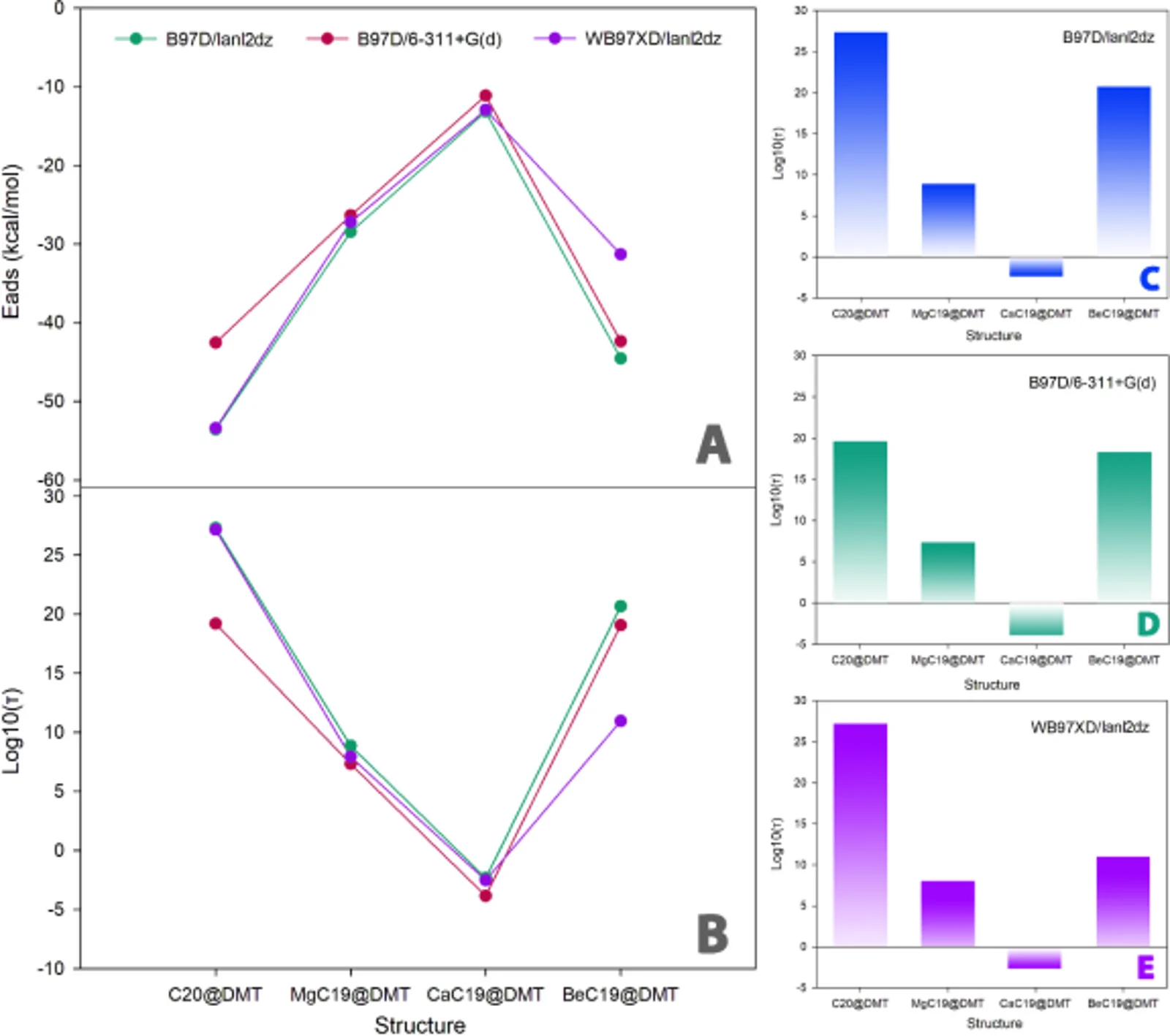
(C, D, and E) (Log_10_(τ), bar representation) further emphasize these trends for each method individually. In all cases, C_20_ and BeC_19_ stand out for significantly larger recovery times, while CaC_19_ consistently shows the lowest values. The agreement across methods highlights the robustness of this conclusion.

In theoretical and computational studies, the emphasis is on qualitative trends rather than absolute numerical values, because the latter can vary with the chosen functional, basis set, and computational approximations. Therefore, the data reported in Table 3 were analyzed qualitatively, and the key trends were visualized in Figs. 8-(A-E) to provide a clearer, more reliable interpretation of the adsorption behavior and sensing performance.

Figure 8-A (Eads) shows how adsorption energies vary across the three computational methods. All methods consistently reproduce the same trend: C_20_ and BeC_19_ exhibit the most negative adsorption energies, followed by MgC_19_, while CaC_19_ shows the weakest interaction. Although the absolute values differ (with ωB97XD/lanl2dz generally predicting fewer negative values for BeC_19_), the qualitative ordering is preserved. This strong agreement confirms that C_20_ and BeC_19_ are the most favorable adsorbents for DMT.

Figure 8-B (Log_10_(τ)) illustrates the recovery time behavior. A clear distinction is observed between strongly and weakly interacting systems. C_20_ and BeC_19_ show extremely high recovery times, whereas MgC_19_ and especially CaC_19_ exhibit much shorter values. Importantly, all three computational methods reproduce the same qualitative pattern, confirming that strong adsorption is directly associated with slow desorption.

Figure 8. (A) Comparison of trends in the adsorption energies (Eads) of DMT on C_20_, BeC_19_, MgC_19_, and CaC_19_, calculated using the B97D/lanl2dz, B97D/6–311 + G(d), and ωB97XD/lanl2dz surfaces; (B) Comparison of recovery times calculated by all three computational methods, expressed as Log_10_(τ); (C-E) Log_10_(τ) bar graphs for each computational method, showing a consistent qualitative trend across methods.

**Fig. 9:**
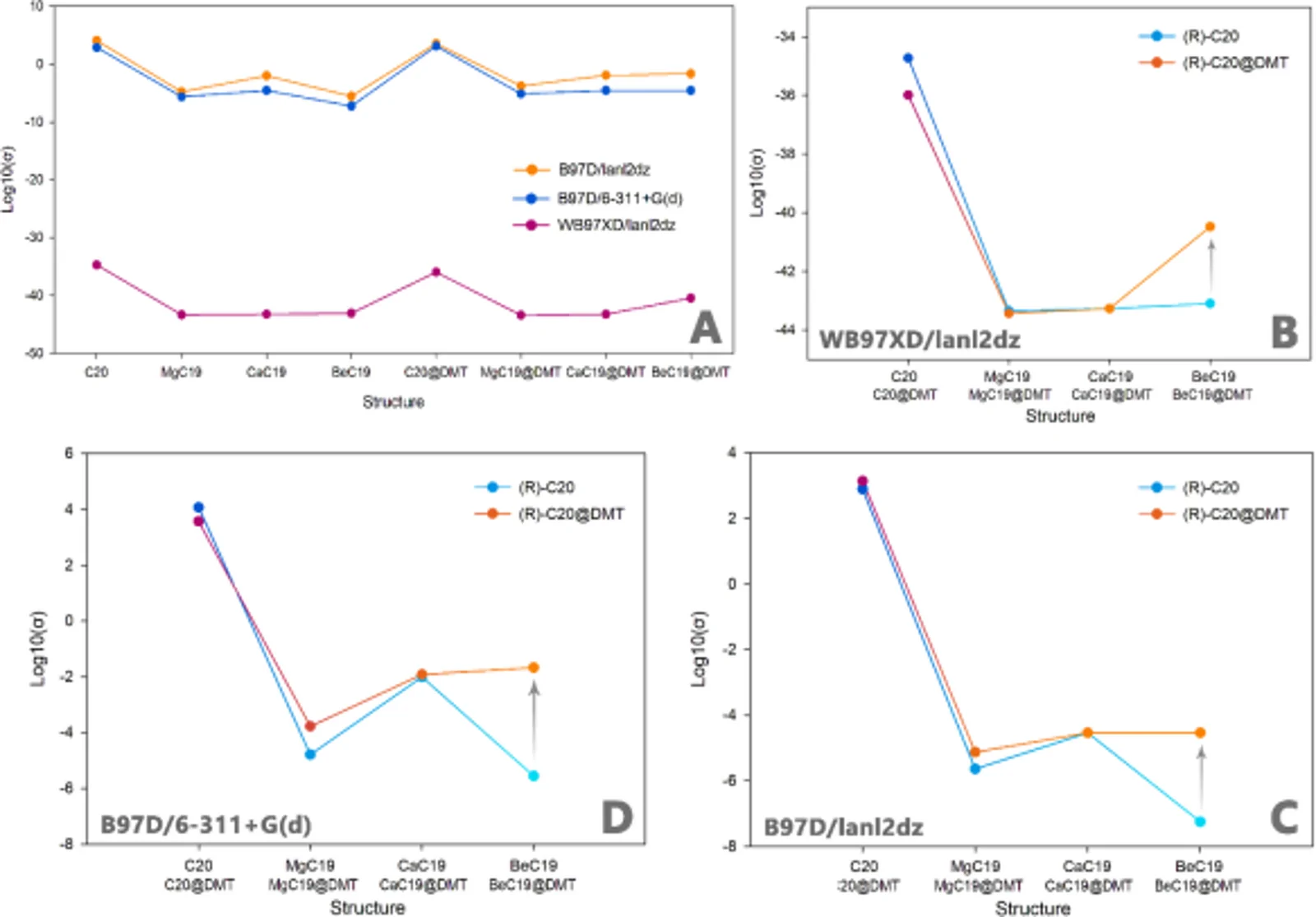
(A) Comparison of the trends in electrical conductivity (Log_10_σ) of the designed structures in the presence or absence of DMT across the three computational levels; (B-D) Comparative plots of post-adsorption conductivity changes for each method, highlighting the significant response of BeC_19_ relative to the other structures.

Figure 9 presents the electrical conductivity analysis using all three computational methods and their overlap, offering more detailed insight into electrochemical sensing behavior. Across all three methods (see Fig. 9-(B, C, and D)), C_20_ shows only moderate changes in conductivity upon adsorption, whereas BeC_19_ exhibits pronounced variation, indicating strong sensitivity to DMT. In contrast, MgC_19_ and CaC_19_ exhibit minimal changes in conductivity, consistent with their weak interaction. These conductivity variations align with the Egap analysis discussed earlier. Systems with smaller Egap (particularly BeC_19_) exhibit greater modulation of electrical conductivity upon DMT adsorption, confirming that band-gap narrowing enhances sensing performance. Importantly, a strong qualitative overlap among B97D/lanl2dz, B97D/6–311 + G(d), and ωB97XD/lanl2dz is observed in all figures (see Fig. 9-A). While the absolute magnitudes differ (particularly for conductivity due to functional-dependent band gap predictions), the relative trends remain unchanged, reinforcing the reliability of the conclusions.

Based on this comprehensive qualitative analysis, both C_20_ and BeC_19_ exhibit strong adsorption energies, indicating their suitability as adsorbents for DMT removal. However, only BeC_19_ shows a significant change in electrical conductivity after adsorption, making it a promising electrochemical sensor. Such long recovery times indicate strong adsorption and slow desorption, which may limit reusability but can be advantageous for capture or filtration applications where irreversible adsorption is desired. Therefore, BeC_19_ is predicted to be a viable option for applications requiring strong adsorption and high sensitivity, but not rapid regeneration, whereas C_19_ is expected to function effectively as an adsorbent without significant sensing capability. These results indicate that BeC_19_ and C_20_ are promising candidates for future experimental validation.

It is important to note that the sensing performance discussed here is specific to DMT and may differ for structurally related molecules such as serotonin and melatonin. Although these compounds share an indole framework, their functional groups differ significantly, leading to variations in electron density distribution, protonation behavior, and interaction sites. For example, serotonin contains a primary amine and a hydroxyl group capable of hydrogen bonding, whereas melatonin contains an amide group with reduced electron-donating ability. In contrast, the tertiary amine in DMT provides stronger donor-acceptor interactions with electron-deficient sites (particularly in BeC_19_), which likely contributes to the enhanced charge transfer and sensing response observed in this study. Therefore, the predicted sensing capability of the studied nanostructures is expected to show selectivity toward DMT, although quantitative assessment of selectivity requires explicit comparative calculations, which will be addressed in future work.

### Dipole moment

The dipole moment is a key descriptor of charge distribution and molecular polarity, and its analysis is especially important in adsorption and sensing studies. An increase in dipole moment upon adsorption typically indicates charge redistribution and enhanced system polarization, which can strengthen electrostatic interactions with the analyte and improve sensitivity in electrochemical sensing^43^. Systems that exhibit significant changes in dipole moment are, therefore, more likely to show measurable electrical responses.

**Table 4:** Dipole moments (Debye) of pristine and DMT-adsorbed C_20_, BeC_19_, MgC_19_, and CaC_19_ structures calculated at the B97D/lanl2dz, B97D/6–311 + G(d), and ωB97XD/lanl2dz levels of theory, illustrating adsorption-induced polarization effects.

Structure	B97D/lanl2dz	B97D/6–311 + G(d)	ωB97XD/lanl2dz
C_20_	0.00	0.00	0.00
MgC_19_	13.19	13.25	15.29
CaC_19_	23.22	19.41	24.47
BeC_19_	4.05	3.73	5.33
C_20_@DMT	18.55	17.12	19.04
MgC_19_@DMT	16.04	15.47	17.73
CaC_19_@DMT	25.66	21.06	27.40
BeC_19_@DMT	12.72	11.37	12.91

**Fig. 10:**
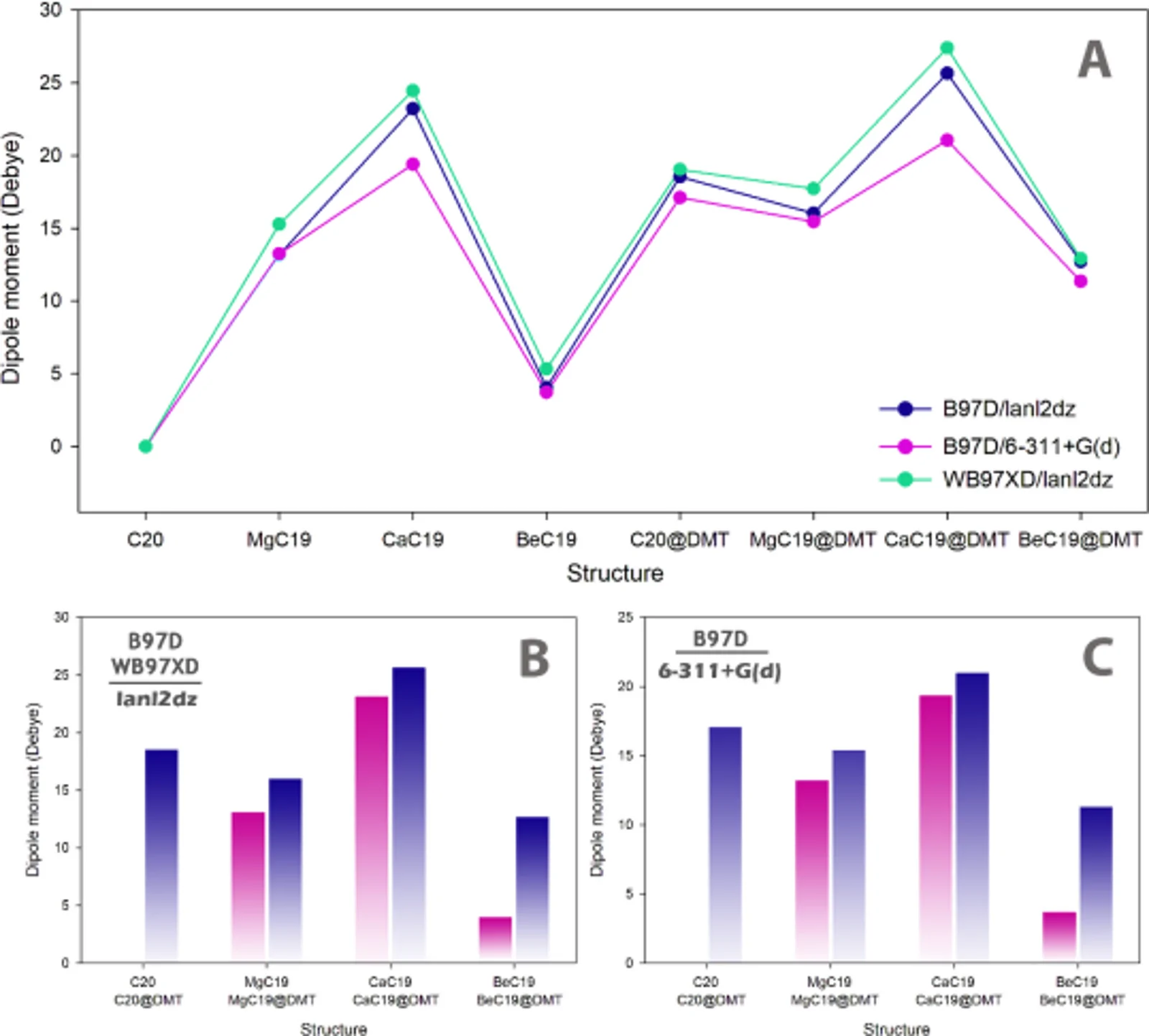
(A) Qualitative comparison of trends in dipole moment changes for pristine and DMT-adsorbed C_20_, BeC_19_, MgC_19_, and CaC_19_ structures, calculated at the B97D/lanl2dz, B97D/6–311 + G(d), and ωB97XD/lanl2dz levels of theory; (B) Qualitative comparison of dipole moments before and after DMT adsorption at B97D/lanl2dz and ωB97XD/lanl2dz; (C) analogous comparison at B97D/6–311 + G(d), highlighting consistent qualitative trends across methods.

Table 4; Fig. 10-A show a consistent qualitative trend across all three computational methods. Pristine C_20_ has a zero-dipole moment (0.00 Debye), reflecting its high symmetry. Upon adsorption, the dipole moment increases substantially (18.55, 17.12, and 19.04 Debye at B97D/lanl2dz, B97D/6–311 + G(d), and ωB97XD/lanl2dz, respectively), indicating strong charge redistribution and DMT-induced polarization. For MgC_19_, the dipole moment is already significant in the pristine state (13.19–15.29 Debye) and increases moderately after adsorption (15.47–17.73 Debye). This suggests some interaction with DMT, but the relative change is modest. In CaC_19_, the highest dipole moments are observed both before and after adsorption (19.41–24.47 Debye for pristine and 21.06–27.40 Debye for the complex). Although the absolute values are large, the relative change upon adsorption is modest, indicating that the system is already highly polarized and less sensitive to additional perturbation. For BeC_19_, the pristine dipole moment is relatively low (3.73–5.33 Debye), but it increases significantly upon adsorption (11.37–12.91 Debye). This marked change reflects strong DMT-induced polarization and suggests a pronounced electronic response.

Importantly, all three computational methods show good qualitative agreement: ωB97XD/lanl2dz predicts slightly higher absolute values due to its treatment of exchange, but the same trends are preserved. Figures 10-B and 10-C further visualize these results, clearly showing the largest dipole increase for C_20_ and BeC_19_, a moderate change for MgC_19_, and minimal relative change for CaC_19_. Overall, the dipole moment analysis supports previous findings: C_20_ and BeC_19_ exhibit the most significant polarization upon DMT adsorption, indicating a strong interaction. In particular, the significant change in the dipole moment of BeC_19_ strengthens our prediction of its potential as an electrochemical sensor, as such polarization changes can directly affect measurable electrical signals.

### UV-Vis Spectrum

The maximum absorption wavelength (λmax) and exciton energy (Eex) are critical parameters for evaluating a structure’s capability as a colorimetric sensor. λmax indicates the wavelength at which the material absorbs light most strongly, providing insight into its response to specific wavelengths, which is essential for detecting certain analytes in a colorimetric sensor. The exciton energy (Eex), on the other hand, represents the energy required to promote an electron to an excited state, which is crucial for understanding the sensor’s optical properties and its ability to undergo the electronic transitions necessary for accurate detection^44^. Each of these parameters has been carefully studied computationally, and the results are shown in Table 5.

**Table 5:** Maximum absorption wavelengths (λmax) and exciton energies (Eex) calculated for the pristine and DMT-adsorbed C_20_, BeC_19_, MgC_19_, and CaC_19_ systems using the B97D and ωB97XD functionals.

Structure	B97D/lanl2dz		ωB97XD/lanl2dz	
	λmax (nm)	Eex (eV)	λmax (nm)	Eex (eV)
C_20_	252	4.90	221	5.59
BeC_19_	319	3.87	230	5.36
MgC_19_	330	3.75	272	4.55
CaC_19_	356	3.47	284	4.36
C_20_@DMT	497	2.49	440	2.87
BeC_19_@DMT	484	2.55	428	2.97
MgC_19_@DMT	409	3.03	294	4.18
CaC_19_@DMT	421	2.93	343	3.61

According to Table 5, all pristine structures absorb in the UV region, with λmax values ranging from 252 to 356 nm (B97D) and 221–284 nm (ωB97XD). Upon DMT adsorption, all systems exhibit a redshift, indicating a modification of the electronic structure and increased delocalization.

Among the structures studied, C_20_ and BeC_19_ show the most pronounced changes. For C_20_, λmax shifts from 252 → 497 nm (B97D) and 221 → 440 nm (ωB97XD), clearly entering the visible region, accompanied by substantial decreases in Eex (4.90 → 2.49 eV and 5.59 → 2.87 eV). Similarly, BeC_19_ shows a strong red shift (319 → 484 nm and 230 → 428 nm) and a notable reduction in Eex. These large changes indicate a strong optical response and high sensitivity to DMT. In contrast, MgC_19_ and CaC_19_ exhibit smaller, less consistent shifts, particularly at the ωB97XD level, indicating weaker optical responsiveness.

Importantly, these optical trends align directly with the adsorption energy results. The systems with the most negative adsorption energies (C_20_ and BeC_19_) also show the largest red shifts and the greatest decrease in Eex. This correlation indicates that stronger adsorption causes greater perturbation of the electronic structure, which in turn enhances the optical response. Conversely, the weaker adsorption observed for MgC_19_ and CaC_19_ is reflected in smaller spectral shifts and reduced sensitivity.

The color change arises from adsorption-induced electronic reorganization. When DMT interacts strongly with the absorber (especially in C_20_ and BeC_19_), significant charge redistribution and orbital interactions narrow the optical gap and reduce the excitation energy. Consequently, electronic transitions require less energy and occur at longer wavelengths, shifting absorption from the UV into the visible region. This shift produces a perceptible color change, forming the basis of colorimetric sensing. In systems with weaker interaction (MgC_19_ and CaC_19_), the electronic structure is only slightly perturbed, resulting in minimal changes in absorption and thus a weaker or negligible colorimetric response.

**Fig. 11:**
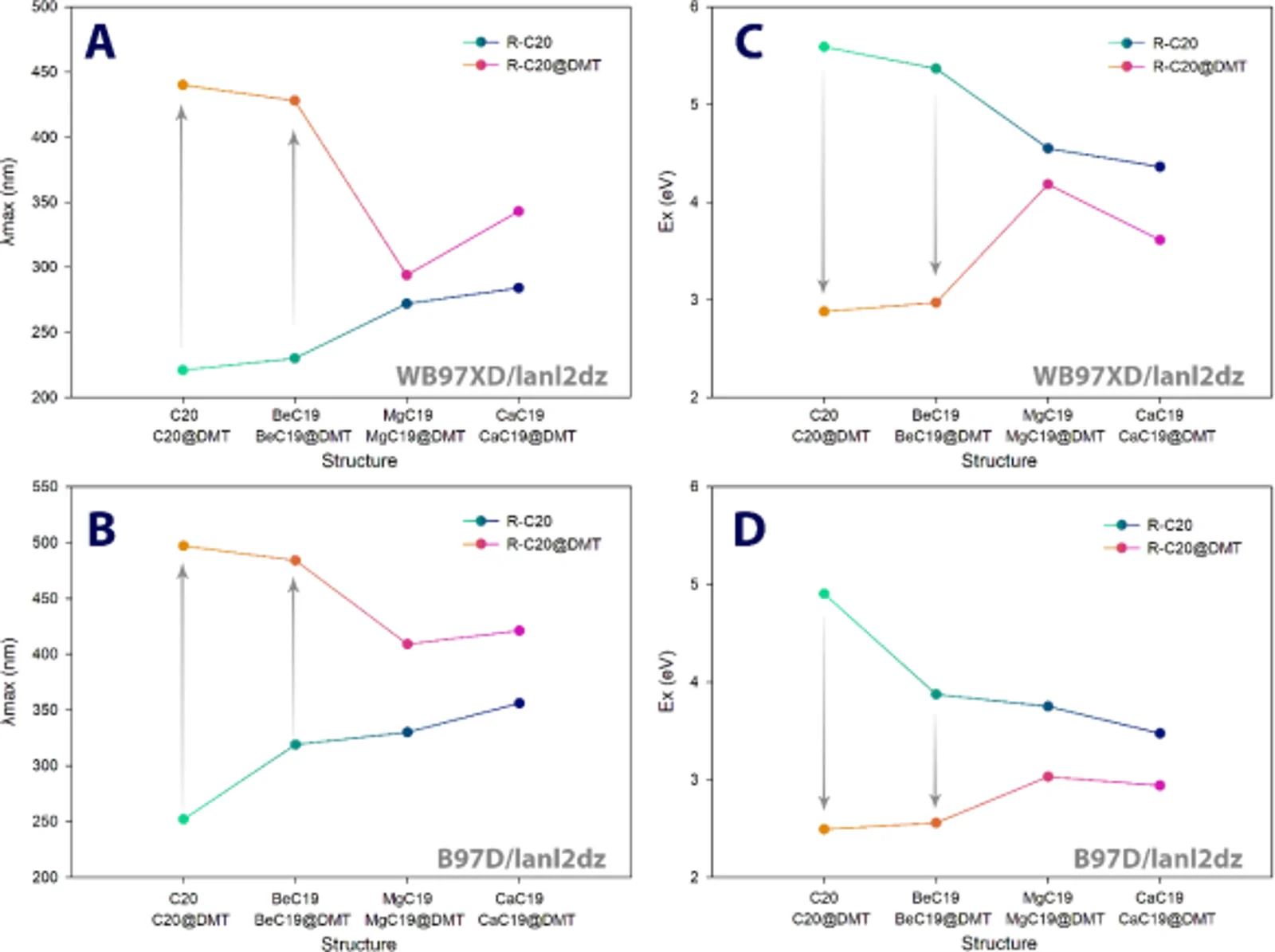
Qualitative comparison of (A-B) absorption maxima (λmax) and (C-D) excitation energies (Eex) for pristine and DMT-adsorbed systems, calculated at the ωB97XD/lanl2dz and B97D/lanl2dz levels, illustrating adsorption-induced red shifts and exciton energy.

As mentioned earlier, because this work is based on theoretical calculations, the emphasis is on qualitative trends and relative changes after adsorption rather than on the absolute values of λmax and Eex, which can vary with the functional group and the chosen basis set. For this purpose, the results presented in Fig. 11 were interpreted within this qualitative framework.

**Fig. 12:**
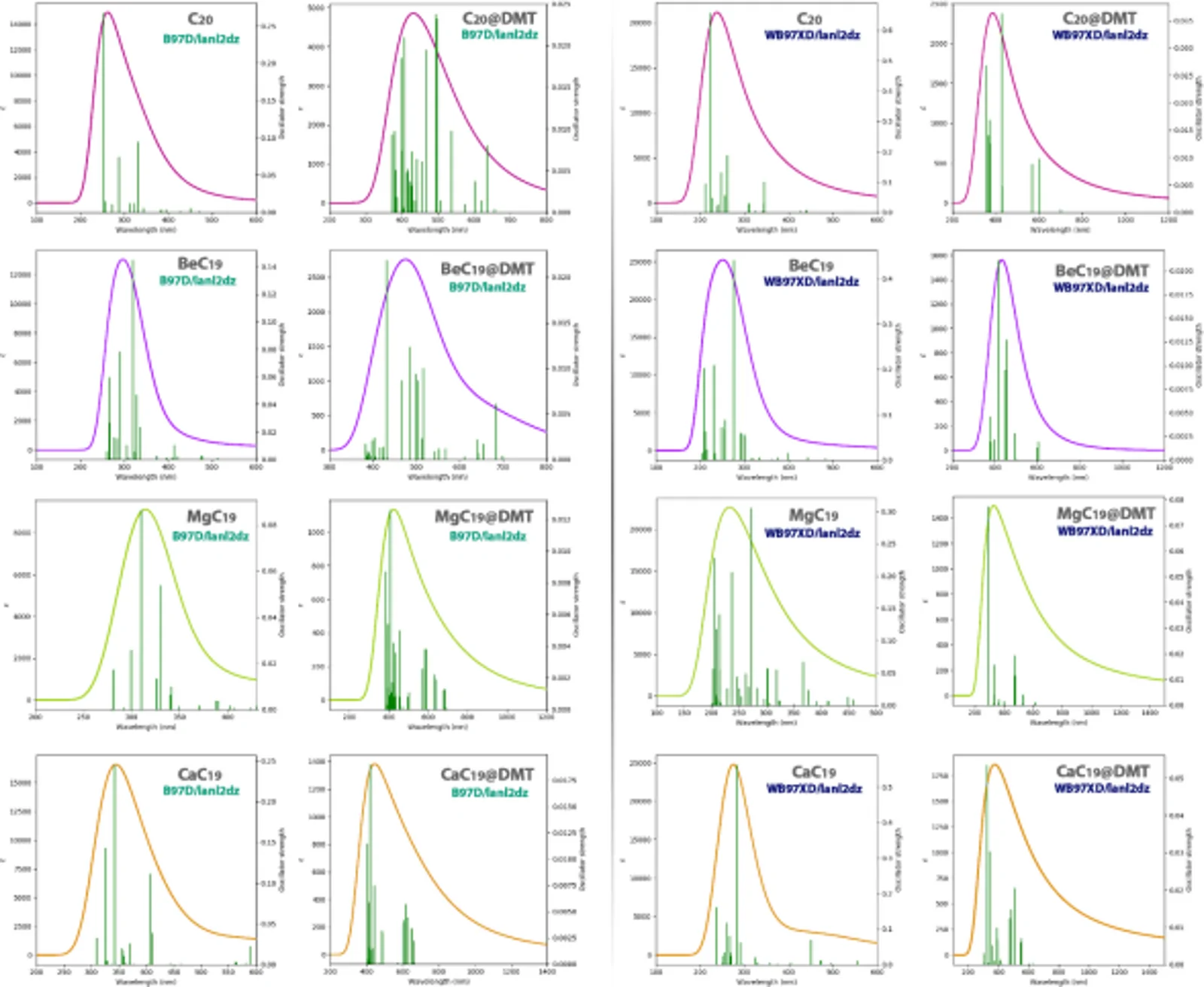
Simulated UV-Vis absorption spectra of the pristine and DMT-adsorbed C_20_, BeC_19_, MgC_19_, and CaC_19_ systems on the B97D/lanl2dz and ωB97XD/lanl2dz surfaces.

In Figs. 11-A and 11-B, after adsorption of DMT, C_20_, and BeC_19_, the λmax shifts from the UV to the visible range, producing a more pronounced color change. This is reflected in the figure by the substantial vertical separation between the pristine (R-C_20_) and adsorbed (R-C_20_@DMT) curves, highlighted by the arrows. Although the absolute λmax values differ between the B97D/lanl2dz and ωB97XD/lanl2dz methods, both panels show the same qualitative behavior: C_20_ and BeC_19_ undergo the largest spectral shifts, while MgC_19_ and CaC_19_ display smaller changes. In Figs. 11-C and 11-D (Eex), the opposite behavior is observed, with a decrease in excitation energy upon adsorption, again most pronounced for C_20_ and BeC_19_. The arrows emphasize this reduction, indicating that less energy is required for electronic excitation after DMT adsorption. As with λmax, the relative reduction at both computational levels follows the same order. Importantly, the correlation between λmax increase and Eex decrease is clearly visible in the figure: systems showing larger red shifts also exhibit greater reductions in excitation energy. This is particularly evident for C_20_ and BeC_19_, confirming stronger electronic perturbation upon adsorption. Finally, the UV spectra of the studied structures are shown in Fig. 12 to provide a clearer visual understanding of changes in λmax in the presence/absence of DMT.

Based on these results, we propose the C_20_ and BeC_19_ structures (due to their acceptable optical responses) as promising candidates for colorimetric sensing of DMT and for further experimental work.

### NCI, RDG, and ELF analysis

**Fig. 13:**
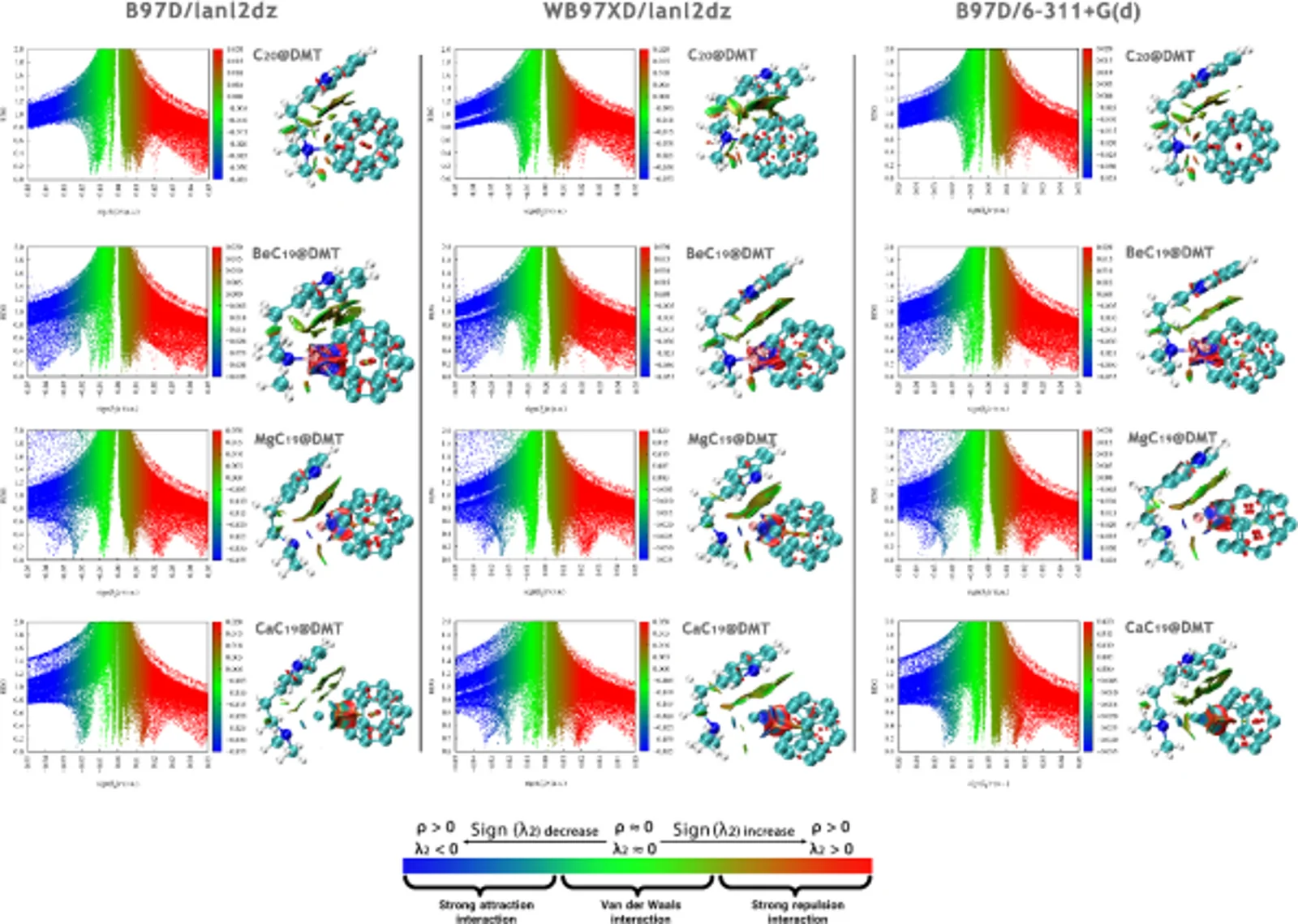
NCI-RDG plots and corresponding isosurfaces for C_20_@DMT, BeC_19_@DMT, MgC_19_@DMT, and CaC_19_@DMT, calculated at the B97D/lanl2dz, ωB97XD/lanl2dz, and B97D/6–311 + G(d) levels, illustrating the nature of intermolecular interactions (blue: attractive, green: van der Waals, red: repulsive).

Investigating noncovalent interactions (NCIs) is crucial for understanding the stability and behavior of molecular systems, especially in complexes where weak forces collectively determine conformational preferences and reactivity. Although each noncovalent force may be weak on its own, their cumulative contribution significantly influences molecular arrangement and the thermodynamic stability of the overall system. Computational NCI analysis provides a reliable means of visualizing these interactions by evaluating the reduced density gradient (RDG) in conjunction with electron density (ρ) and the sign of the second eigenvalue of the electron density Hessian matrix [sign(λ_2_)ρ]. This approach identifies and distinguishes attractive, repulsive, and dispersive regions within the molecular framework. Figure 13 presents NCI scatter plots and the corresponding isomer levels for each of the studied complexes. The color mapping (blue-green-red) clearly differentiates strong attractive forces (blue), weak van der Waals interactions (green), and steric repulsion (red). This analysis offers valuable insight into how molecular geometry modulates the nature and spatial distribution of these noncovalent forces, thereby affecting the overall electronic environment and stability of each conformer^45,46^.

Figure 13 presents the noncovalent interaction (NCI) analysis of DMT complexes with C_20_, BeC_19_, MgC_19_, and CaC_19_ at three computational levels (B97D/lanl2dz, ωB97XD/lanl2dz, and B97D/6–311 + G(d)). Importantly, all three methods show very similar qualitative patterns, confirming the robustness and reliability of the results. For the C_20_@DMT complex, the interaction is characterized by broad, green equivalent surfaces distributed over a large contact area between the cage and the DMT molecule. This indicates that adsorption is dominated by van der Waals interactions. However, the large spatial extent of these interactions indicates a significant aggregation effect, which explains the very high adsorption energy observed for this system. Thus, although the interaction is not highly localized, its overall strength is significant due to the large interface.

For BeC_19_@DMT, the NCI analysis reveals a distinct interaction pattern. In addition to the green regions, distinct blue spots appear near the Be atom and the DMT molecule’s interacting region, indicating strong, localized attractive interactions. These regions are clearly visible across all computational methods and show that the Be impurity introduces a more specific and stronger interaction site. The coexistence of these strong localized interactions with the surrounding van der Waals contributions explains the high adsorption energy of BeC19, which is comparable to that of C_20_.

For MgC_19_@DMT, the interaction is weaker and more diffuse. The NCI coplanar surfaces are predominantly green, with only minor blue contributions, indicating that adsorption is governed mainly by weak van der Waals interactions with limited attractive specificity. This observation is consistent with the average adsorption energy reported for this system. A similar behavior is observed for CaC_19_@DMT, where the interaction is dominated by green regions and lacks significant blue features. This indicates weak, non-specific interactions between the cage and DMT. As a result, CaC_19_ shows the lowest adsorption energy among the studied systems. According to the results, the strong and/or extensive attractive interactions in C_20_ and BeC19 are justified by their high adsorption energies, whereas MgC_19_ and CaC_19_, which show lower adsorption power in the presence of DMT, exhibit weak van der Waals interactions.

ELF analysis is useful because it visualizes the localization of electron density within the adsorbent@DMT complexes. In adsorption studies, this helps clarify whether the interaction region involves strong localized electron accumulation or primarily diffuse, noncovalent electron redistribution.

**Fig. 14:**
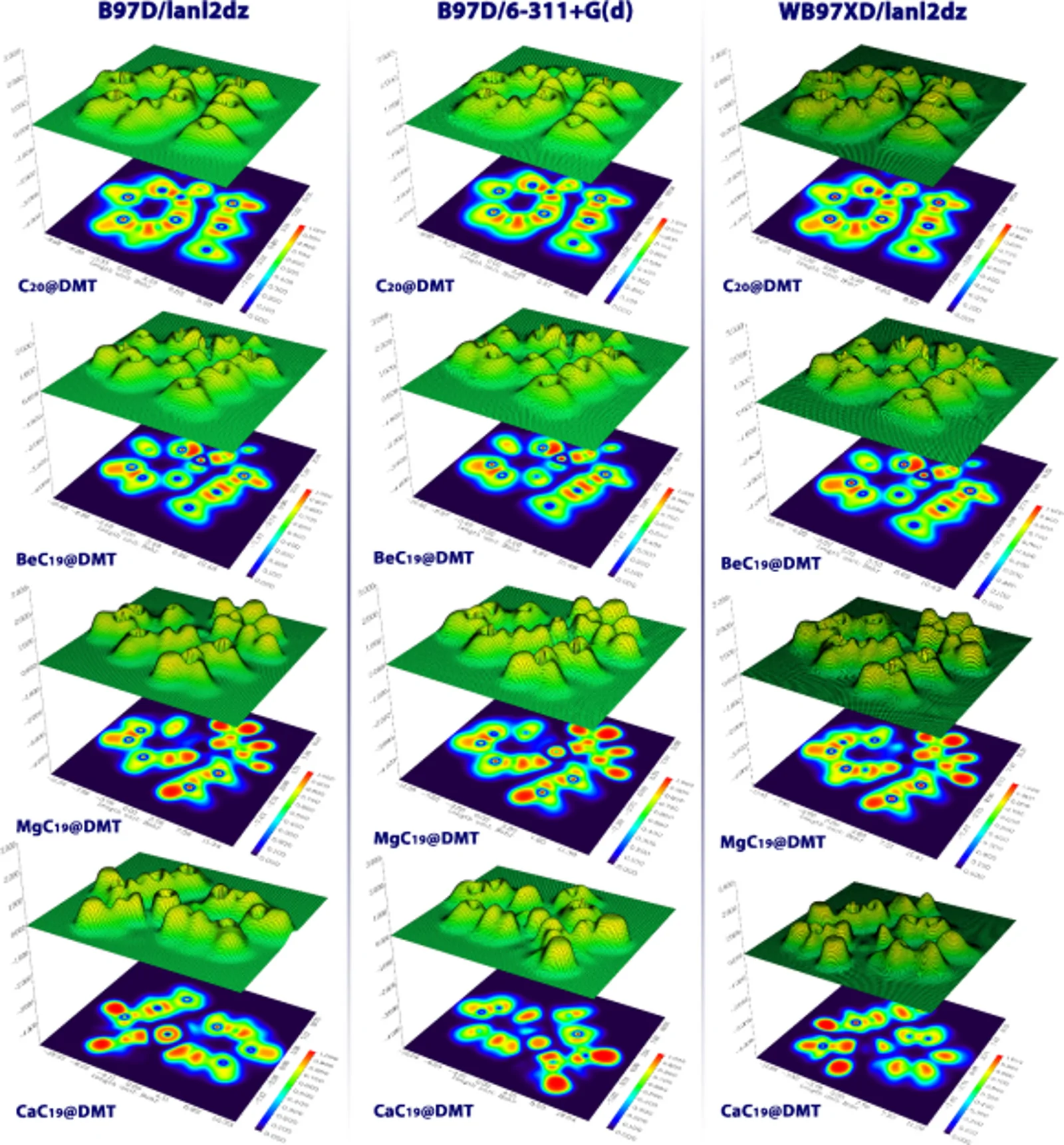
3D electron localization function (ELF) surfaces and contour maps for C_20_@DMT, BeC_19_@DMT, MgC_19_@DMT, and CaC_19_@DMT, calculated at the B97D/lanl2dz, B97D/6–311 + G(d), and ωB97XD/lanl2dz levels, showing electron localization and charge redistribution due to adsorption.

The ELF maps show good qualitative agreement with the NCI results. For C_20_@DMT, the ELF distribution is primarily localized on the C_20_ cage and the DMT fragments separately, with no clear continuous localization basin between them. This supports the NCI interpretation that adsorption is dominated by extended noncovalent interactions distributed over a broad contact surface rather than by a highly localized bond-like interaction. The large adsorption energy of C_20_@DMT can therefore be attributed to the cumulative contribution of many weak interactions.

For BeC_19_@DMT, the ELF contours show the most pronounced electron redistribution near the Be-doped region and the interacting part of DMT. Although a fully covalent-type localization basin is not clearly formed, the enhanced localization and polarization around the Be···DMT contact are consistent with the blue attractive regions observed in the NCI plots. This confirms that BeC_19_ provides a more localized and stronger interaction site for DMT.

In MgC_19_@DMT, the ELF pattern remains largely distinct between the adsorbent and DMT, with only weak localization changes at the interface. This aligns with the NCI results, which show the interaction primarily as green van der Waals regions with limited attractive character. Thus, MgC_19_ exhibits a moderate, not strongly localized, interaction with DMT.

For CaC_19_@DMT, the ELF maps show diffuse, weak interfacial localization, with electron density primarily retained on the individual fragments. This is consistent with the NCI analysis, which indicated predominantly weak van der Waals interactions and minimal strong attractive features. Accordingly, CaC_19_ exhibits the weakest adsorption among the studied complexes.

Overall, the ELF results reinforce the NCI conclusions: C_20_@DMT is stabilized primarily by extensive weak interactions, BeC1_9_@DMT by more localized attractive interactions around the Be site, and MgC_19_@DMT and CaC_19_@DMT by weaker, more diffuse interactions (Fig. 14).

**Fig. 15:**
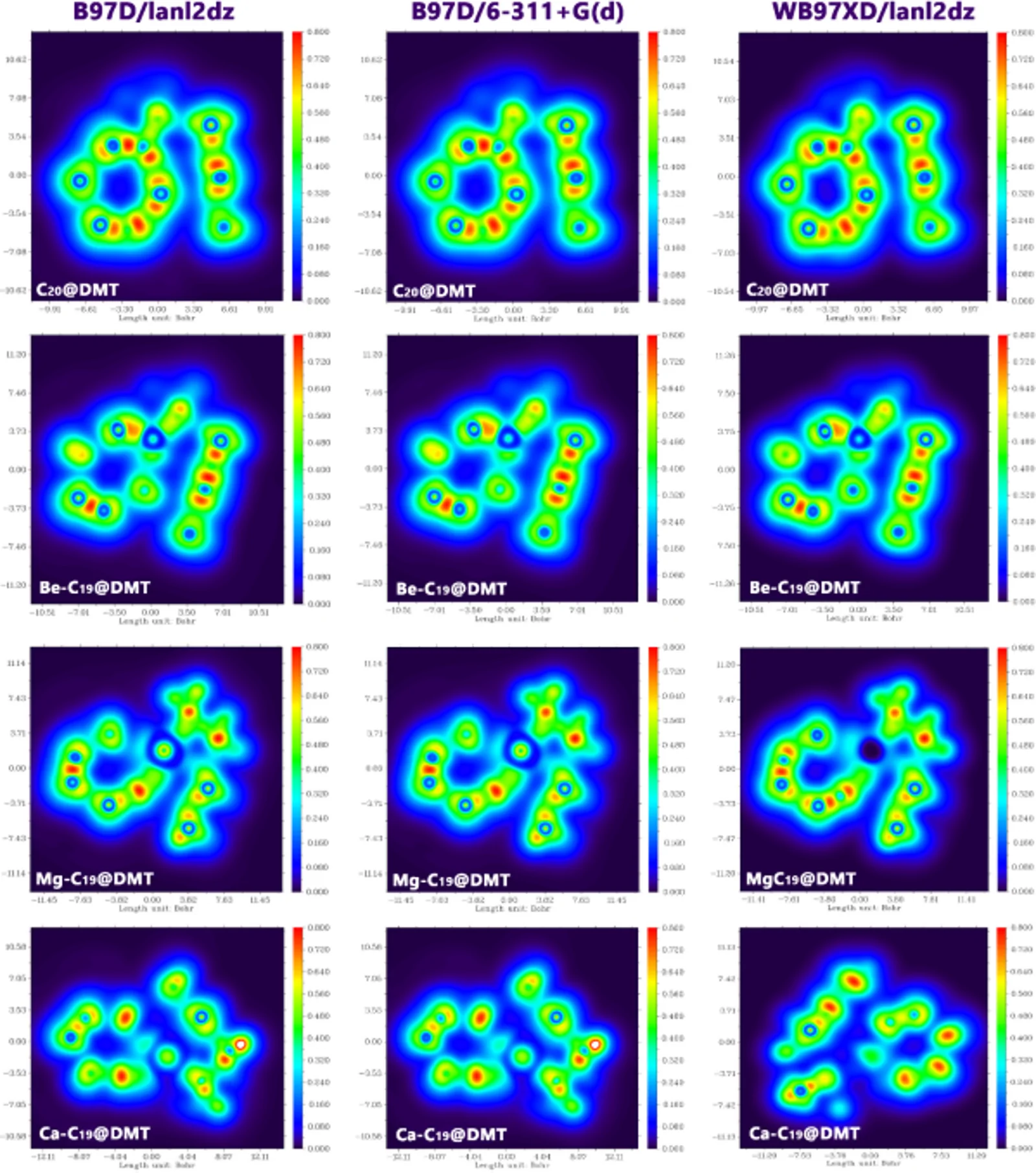
Localized Orbital Locator (LOL) contour maps for C_20_@DMT, BeC_19_@DMT, MgC_19_@DMT, and CaC_19_@DMT, calculated at the B97D/lanl2dz, B97D/6–311 + G(d), and ωB97XD/lanl2dz levels, illustrating electron localization and interfacial charge distribution upon adsorption.

The Localized Orbital Locator (LOL) contours provide valuable insight into the extent of electron localization and delocalization within the studied complexes, complementing ELF and NCI analyses. High LOL values (red/yellow regions) correspond to strongly localized electrons associated with bonds or lone pairs, whereas low values (blue/green regions) indicate delocalized electron density and weak interactions. In the context of adsorption, LOL is particularly useful for determining whether the interaction leads to the formation of new localized bonding regions or remains predominantly noncovalent and diffuse Fig. 15.

A consistent qualitative pattern emerges across all three computational methods (B97D/lanl2dz, B97D/6–311 + G(d), and ωB97XD/lanl2dz), confirming the reliability of the results. For the C_20_@DMT complex, the LOL contours show strong localization around the carbon cage and within the DMT molecule, while the interfacial region between them remains relatively low in localization. This indicates that no new localized bond forms upon adsorption, and the interaction is governed mainly by delocalized, noncovalent forces distributed over a large surface area. This observation is fully consistent with the NCI results, in which extended van der Waals interactions were dominant.

For BeC_19_@DMT, a noticeable enhancement in electron localization is observed near the Be atom and in the interacting region of DMT. Although a fully continuous, high-LOL bridge is not formed, the increased localization in this region indicates stronger polarization and a more directed interaction than in the other systems. This behavior aligns with the NCI analysis, which revealed distinct attractive interactions around the Be site, and explains the relatively high adsorption energy of this complex.

For MgC_19_@DMT, the LOL contours are largely similar to those of C20@DMT, with electron localization primarily confined to the individual fragments. The interaction region shows only weak localization, indicating that adsorption is dominated by diffuse van der Waals interactions with limited electronic coupling. A similar trend is observed for CaC_19_@DMT, where the LOL distribution remains largely separated between the cage and DMT, with minimal localization in the interfacial region. This confirms that the interaction is weak and non-specific, consistent with the NCI analysis and the system’s low adsorption energy.

Overall, the LOL results show no clear evidence of fully developed covalent bond formation, and the interactions are predominantly non-covalent, with minor covalent character in the Be-doped system. However, the degree of electron localization and polarization varies among the systems, following the trend BeC_19_ > C_20_ > MgC_19_ ≈ CaC_19_. This trend is consistent with the observed significant adsorption energies and supports the conclusion that BeC_19_ and C_20_ exhibit stronger interactions with DMT, whereas MgC_19_ and CaC_19_ show weaker interactions.

### QTAIM analysis

**Fig. 16:**
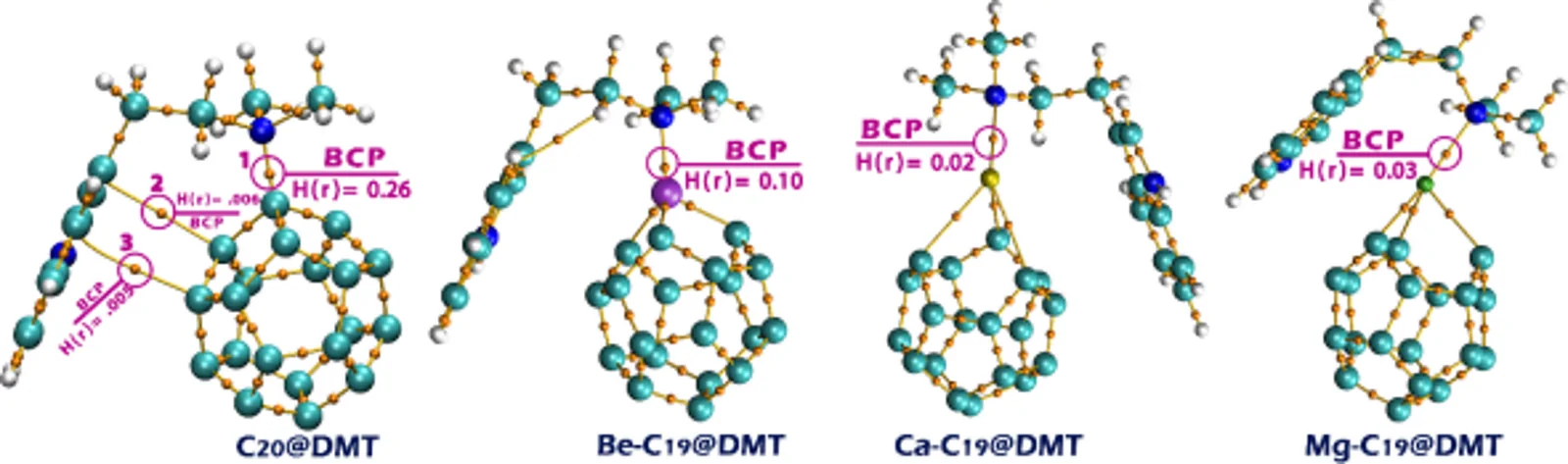
H(r) values at the bond critical points (BCPs) for each of the C_20_@DMT, BeC_19_@DMT, CaC_19_@DMT, and MgC_19_@DMT structures.

Quantum Theory of Atoms in Molecules (QTAIM) analysis was used to characterize the nature and strength of interactions between DMT and the studied nanostructures by examining the topological properties at the bond critical points (BCPs) (Fig. 16). In QTAIM, the electron density at the BCP, ρ(r), is a primary indicator of interaction strength, with larger values corresponding to stronger interactions. The Laplacian of the electron density, ∇^2^ρ(r), provides insight into the nature of bonding: negative values indicate shared-shell (covalent) interactions, whereas positive values correspond to closed-shell interactions such as electrostatic, hydrogen bonding, or van der Waals forces. In addition, the kinetic energy density G(r) and potential energy density V(r) were used to evaluate the total energy density, defined as H(r) = G(r) + V(r). The sign of H(r) further distinguishes interaction types: H(r) < 0 suggests partial covalent character, while H(r) > 0 indicates noncovalent interactions. Moreover, the ratio |V(r)|/G(r) serves as a useful descriptor, where values less than 1 correspond to closed-shell interactions and values greater than 1 indicate shared-shell (covalent) interactions^47^.

**Table 6:** QTAIM parameters at the bond critical points (BCPs) for the C_20_@DMT, BeC_19_@DMT, MgC_19_@DMT, and CaC_19_@DMT complexes, calculated at the B97D/6–311 + G(d) level of theory, including electron density (ρ(r)), Laplacian of the electron density (∇^2^ρ(r)), kinetic energy density (G(r)), potential energy density (V(r)), total energy density (H(r) = G(r) + V(r)), and the ratio |V(r)|/G(r). All values are in a.u.

Structure		ρ(*r*)	∇^2^ρ(*r*)	G(*r*)	V(*r*)	|V(*r*)|/G(*r*)
C_20_@DMT	BCP 1	0.2048	0.0895	0.0872	0.1767	2.026
	BCP 2	0.0143	−0.0098	0.0082	−0.0015	0.182
	BCP 3	0.0119	−0.0091	0.0074	−0.0017	0.229
MgC_19_@DMT		0.0338	−0.0475	0.0422	−0.0053	0.125
CaC_19_@DMT		0.0271	−0.0252	0.0233	−0.0018	0.077
BeC_19_@DMT		0.0674	−0.0888	0.0973	0.0085	0.087

The calculated QTAIM parameters for all complexes are summarized in Table 6. For the C20@DMT system, three BCPs were identified, indicating that DMT interacts with the fullerene surface at multiple contact points. Among these, BCP 1 exhibits a higher electron density (ρ = 0.2048 a.u.), suggesting a stronger local interaction than at the other contact points. However, the corresponding Laplacian is positive (∇^2^ρ = 0.0895 a.u.), indicating that this interaction remains within the closed-shell regime. The additional BCPs exhibit much lower electron densities (ρ ≈ 0.01 a.u.), confirming that the overall stabilization arises from the cumulative contribution of multiple weak interactions rather than from the formation of a covalent bond. This observation is fully consistent with the NCI and ELF analyses, which indicated extended van der Waals interactions over a broad contact surface.

For the BeC_19_@DMT complex, the BCP shows a higher electron density (ρ = 0.0674 a.u.) than in the MgC_19_ and CaC_19_ systems, along with a more pronounced Laplacian. This suggests a stronger, more localized interaction between DMT and the Be-doped cage. Nevertheless, the values remain within the range typical of noncovalent closed-shell interactions, indicating that the interaction is dominated by electrostatic attraction and polarization, possibly with a degree of donor-acceptor character involving the nitrogen lone pair and the electron-deficient Be center.

In contrast, the MgC_19_@DMT and CaC_19_@DMT complexes exhibit significantly lower electron densities at the BCPs (ρ = 0.0338 and 0.0271 a.u., respectively) and small Laplacian values, confirming weak, diffuse interactions. These features are characteristic of van der Waals-dominated adsorption with minimal electronic coupling between the adsorbent and the analyte. The weaker interaction strength observed for these systems is consistent with their lower adsorption energies and the minimal perturbation of electronic properties discussed in previous sections.

Overall, the combined analysis of ρ(r), ∇^2^ρ(r), and energy density criteria shows that no true covalent bond forms in any of the studied complexes. All methods consistently indicate that the adsorption is non-covalent. The adsorption process is therefore governed primarily by closed-shell interactions, including electrostatic, polarization, and dispersion forces. The relative interaction strength follows the trend: C_20_@DMT > BeC_19_@DMT > MgC_19_@DMT ≈ CaC_19_@DMT. This trend is in excellent agreement with the adsorption energy, NCI, ELF, and LOL analyses, which show that these interactions remain within the closed-shell range and are influenced by dispersion, electrostatic, and polarization effects, confirming the reliability and consistency of the results across different theoretical approaches.

### Validation of computational methods and reported results

To ensure the reliability and consistency of the computational results from the three theoretical approaches (B97D/lanl2dz, B97D/6–311 + G(d), and ωB97XD/lanl2dz), a statistical validation was performed using the mean absolute deviation (MAD), root-mean-square deviation (RMSD), and Pearson correlation coefficient (R)^48^. These metrics are widely used to assess agreement between datasets and are particularly important in theoretical studies, where absolute values may vary due to methodological approximations. The mean absolute deviation (MAD) provides a direct measure of the average difference between two sets of calculated values obtained at different computational levels. It reflects how much one method deviates from another on average, without considering the direction of the deviation. A small MAD indicates that the methods yield numerically similar results, while larger values suggest greater discrepancies. In this study, MAD was used to compare the results obtained from the ωB97XD/lanl2dz, B97D/lanl2dz, and B97D/6–311 + G(d) methods.

For a set of N corresponding values, x_i_ (ωB97XD/lanl2dz) and y_i_ (B97D/lanl2dz or B97D/6–311 + G(d)), the mean absolute deviation (MAD) is defined as (Eq. 11):

11 $$\:\boldsymbol{M}\boldsymbol{A}\boldsymbol{D}=\frac{1}{\boldsymbol{N}}\sum\:_{\boldsymbol{i}=1}^{\boldsymbol{N}}\left|{\boldsymbol{x}}_{\boldsymbol{i}}-{\boldsymbol{y}}_{\boldsymbol{i}}\right|$$

which measures the average magnitude of the absolute differences between the two computational approaches^49^.

The root-mean-square deviation (RMSD) provides a more sensitive evaluation of deviations by giving greater weight to larger differences (Eq. 12). This parameter is particularly useful for determining whether agreement between methods is consistent across all data points or is influenced by occasional large deviations. Low RMSD values indicate that the calculated properties are closely grouped, whereas higher values may indicate outliers.

The RMSD is calculated as:

12 $$\:\boldsymbol{R}\boldsymbol{M}\boldsymbol{S}\boldsymbol{D}=\sqrt{\frac{1}{\boldsymbol{N}}\sum\:_{\boldsymbol{i}=1}^{\boldsymbol{N}}{\left({\boldsymbol{x}}_{\boldsymbol{i}}-{\boldsymbol{y}}_{\boldsymbol{i}}\right)}^{2}}$$

The Pearson correlation coefficient (R) quantifies the strength and direction of the linear relationship between two datasets (Eq. 13). Unlike MAD and RMSD, which focus on numerical differences, R assesses whether the overall trends predicted by different methods are consistent. Values of R near + 1 indicate a strong positive correlation, meaning the methods predict the same variation pattern across the studied systems.

The Pearson correlation coefficient (R) is given by:

13 $$\:\boldsymbol{R}=\sqrt{\frac{{\sum\:}_{\boldsymbol{i}=1}^{\boldsymbol{N}}\left({\boldsymbol{x}}_{\boldsymbol{i}}-\stackrel{-}{\boldsymbol{x}}\right)\left({\boldsymbol{y}}_{\boldsymbol{i}}-\stackrel{-}{\boldsymbol{y}}\right)}{{\sum\:}_{\boldsymbol{i}=1}^{\boldsymbol{N}}{\left({\boldsymbol{x}}_{\boldsymbol{i}}-\stackrel{-}{\boldsymbol{x}}\right)}^{2}{\sum\:}_{\boldsymbol{i}=1}^{\boldsymbol{N}}{\left({\boldsymbol{y}}_{\boldsymbol{i}}-\stackrel{-}{\boldsymbol{y}}\right)}^{2}}}$$

Here, xi represents the value of a given calculated parameter obtained at the ωB97XD/lanl2dz level of theory for system i, while yi denotes the corresponding value computed for the same system using either B97D/lanl2dz or B97D/6–311 + G(d). Thus, each pair (x_i_,y_i_) corresponds to identical physical quantities evaluated for the same molecular structure using different functionals and/or basis sets. The terms $\:\stackrel{-}{x}$ and $\:\stackrel{-}{y}$ represent the mean values of the respective datasets^50^.

**Table 7:** Statistical comparison (MAD, RMSD, and Pearson correlation coefficient R) between computational methods.

Parameter	Comparison	MAD	RMSD	*R*
Egap	B97D/lanl2dz vs. B97D/6–311 + G(d)	0.200	0.223	0.976
Egap	B97D/lanl2dz vs. ωB97XD/lanl2dz	4.658	4.659	0.941
Eads	B97D/lanl2dz vs. B97D/6–311 + G(d)	4.335	5.812	0.978
Eads	B97D/lanl2dz vs. ωB97XD/lanl2dz	3.723	6.651	0.934
σ (log scale)	B97D/lanl2dz vs. B97D/6–311 + G(d)	1.690	1.887	0.976
σ (log scale)	B97D/lanl2dz vs. ωB97XD/lanl2dz	39.441	39.461	0.932
τ (log scale)	B97D/lanl2dz vs. B97D/6–311 + G(d)	3.183	4.265	0.978
τ (log scale)	B97D/lanl2dz vs. ωB97XD/lanl2dz	2.740	4.883	0.934
Dipole moment	B97D/lanl2dz vs. B97D/6–311 + G(d)	1.518	2.235	0.991
Dipole moment	B97D/lanl2dz vs. ωB97XD/lanl2dz	1.093	1.312	0.997
λmax	B97D/lanl2dz vs. ωB97XD/lanl2dz	69.50	73.42	0.955
Eex	B97D/lanl2dz vs. ωB97XD/lanl2dz	0.813	0.883	0.941

**Fig. 17:**
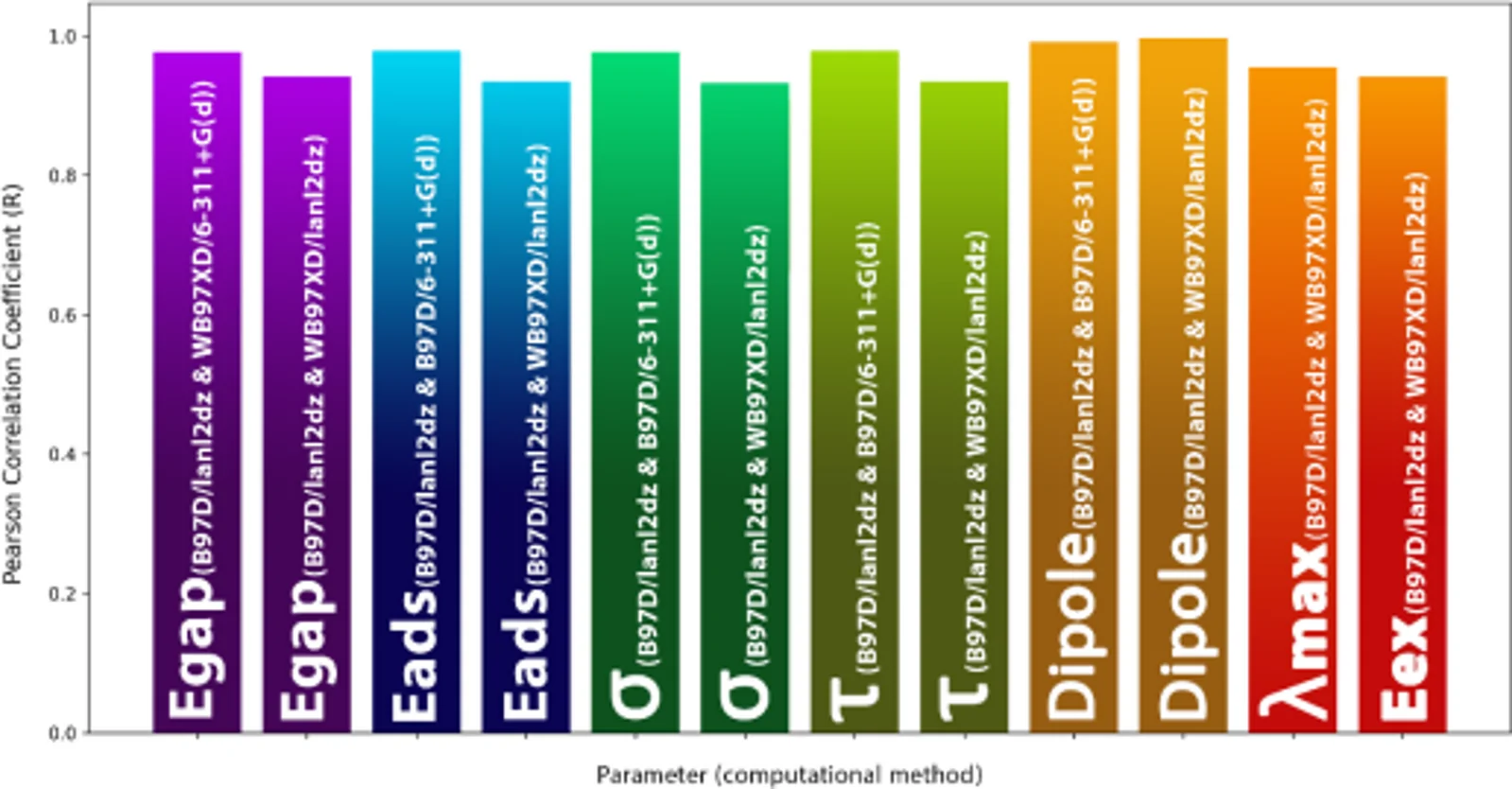
Statistical validation of the computational methods using the Pearson correlation matrix to compare the results of B97D/lanl2dz, B97D/6–311 + G(d), and ωB97XD/lanl2dz for the most important computed descriptors.

The statistical analysis in Table 7 offers a comprehensive assessment of the consistency and reliability of the three computational approaches used in this work. Overall, the results show strong agreement among the methods, particularly when comparing B97D/lanl2dz with B97D/6–311 + G(d). This is evident in the low MAD and RMSD values and the very high Pearson correlation coefficients (*R* ≈ 0.97–0.99) for most properties, including Egap, Eads, electrical conductivity (σ), recovery time (τ), and dipole moment. These high correlations confirm that using different basis sets within the same functional framework does not significantly alter qualitative trends, indicating good basis-set convergence and the robustness of the calculated descriptors.

Compared with ωB97XD/lanl2dz, B97D/lanl2dz shows slightly larger deviations, as evidenced by higher MAD and RMSD values. This difference is expected because the ωB97XD functional includes long-range exact exchange, which systematically affects properties such as Egap and σ. Despite these quantitative differences, the Pearson correlation coefficients remain high (*R* ≈ 0.93–0.95 for most properties), confirming that the relative trends are preserved across functionals (Fig. 17). Notably, the dipole moment shows the highest level of agreement (*R* = 0.997), indicating that polarization effects are consistently described regardless of the computational method. Similarly, optical properties (λmax and Eex) exhibit a strong correlation (*R* ≈ 0.94–0.96), supporting the reliability of the predicted colorimetric behavior.

The largest deviations are observed for electrical conductivity, particularly when comparing B97D with ωB97XD, where high MAD and RMSD values arise from the exponential dependence of conductivity on the energy gap. Nevertheless, the high correlation coefficient (*R* = 0.932) indicates that the trend in conductivity changes upon adsorption is still well preserved. A similar observation holds for recovery time, where deviations are moderate in magnitude, but the overall ranking of adsorption strength and desorption behavior remains consistent.

Overall, the statistical metrics clearly show that although absolute values vary with the computational method, the qualitative trends are highly reliable and method-independent. This is crucial for theoretical studies, as it confirms that conclusions about adsorption strength, sensing performance, and optical response are not artifacts of a specific functional or basis set. Based on these findings, the results of this work are suitable for future experimental validation. Therefore, future experimental efforts can strategically focus on the structures designed in this study, and it is anticipated that the results of this work can reduce trial-and-error approaches and ultimately accelerate the development of practical measurement platforms.

### Future Work

While the present study provides a comprehensive computational investigation of the adsorption and sensing behavior of dimethyltryptamine (DMT) on pristine and metal-doped C_20_ fullerenes, it is important to emphasize that no pharmacokinetic or toxicological evaluations were performed. Therefore, extending these findings toward pharmaceutical or biomedical applications remains beyond the scope of the current work. Future studies may incorporate advanced in silico approaches to evaluate the biological safety and pharmacological relevance of these systems, as shown in previous studies that used computational methods to assess biological interactions and safety profiles^51^. In particular, absorption, distribution, metabolism, excretion, and toxicity (ADMET) analyses could be employed to assess key pharmacokinetic properties. Additionally, cardiotoxicity prediction methods such as Pred-hERG analysis would be valuable for evaluating potential inhibitory effects on the human ether-à-go-go-related gene (hERG) potassium channel, a critical parameter in drug safety assessment. Furthermore, computational toxicology tools could be used to estimate the mutagenicity, cytotoxicity, and long-term biological effects of both analyte–nanostructure complexes and doped fullerene systems. Such analyses would provide a more complete understanding of the potential risks and limitations associated with their use in biological environments. In this context, previously reported methodologies for ADMET and toxicity prediction may serve as a useful framework for extending the present work toward pharmaceutical applications. Integrating these approaches with the current adsorption and sensing results would enable a more holistic evaluation of the designed nanostructures and support their potential translation into practical biomedical or forensic technologies^52,53^. Overall, incorporating ADMET, Pred-hERG, and toxicological assessments represents a critical next step for validating the suitability of these materials beyond sensing applications and for exploring their broader applicability in the pharmaceutical domain.

## Conclusion

In this comprehensive computational study, the adsorption and sensing behavior of dimethyltryptamine (DMT) on pristine C_20_ fullerene and its beryllium (Be), magnesium (Mg), and calcium (Ca)-doped derivatives were systematically investigated using density functional theory (DFT), time-dependent DFT (TD-DFT), and quantum theory of atoms in molecules (QTAIM). Three complementary computational methods (B97D/lanl2dz, B97D/6–311 + G(d), and ωB97XD/lanl2dz) were employed to ensure methodological robustness and to validate the observed qualitative trends. This work is intended as a pre-synthesis computational investigation aimed at introducing suitable candidates for future experimental work, thereby reducing the time, cost, and trial-and-error associated with laboratory efforts. Consequently, the reported numerical values should not be considered as absolute or as replacements for experimental measurements; rather, they provide qualitative insights and relative trends that require future experimental validation.

Structural analysis showed that doping Be, Mg, and Ca into the C_20_ cage caused significant changes in the structural and electronic properties of pristine C_20_. Adsorption energy calculations showed that pristine C_20_ and BeC_19_ have stronger interactions (chemisorption) with DMT than those of other structures (MgC_19_ and CaC_19_). The stronger adsorption in C20 and BeC_19_ results from distinct mechanisms: C_20_@DMT is stabilized by extensive van der Waals interactions distributed over a large contact surface, while BeC_19_@DMT benefits from a more localized donor-acceptor interaction at the electron-acceptor center and acceptor-donor center at the more electron-acceptor center.

Electronic structure analysis revealed that BeC_19_ and C_20_ undergo the greatest perturbation, with a significant reduction in the energy gap in the presence of DMT (and consequently significant changes in other solvation parameters). In contrast, MgC_19_ and CaC_19_ showed minimal changes in Egap and negligible charge transfer. Density of states (DOS), total density of states (TDOS), and predicted density of states (PDOS) analyses confirmed these trends, showing significant redistribution near the Fermi level for BeC_19_ and C_20_. At the same time, MgC_19_ and CaC_19_ remained largely unaffected. Frontier molecular orbital analysis demonstrated clear spatial separation in BeC_19_@DMT, with the HOMO localized on DMT and the LUMO concentrated on the Be-doped cage, confirming a donor-acceptor mechanism. Conversely, MgC_19_@DMT and CaC_19_@DMT showed both the HOMO and LUMO predominantly localized on the cage, indicating weak orbital overlap.

Electrical conductivity analysis showed that BeC_19_ exhibits a significant increase (approximately a fourfold increase) after DMT adsorption. C_20_ showed a modest decrease in conductivity, while MgC_19_ and CaC_19_ showed negligible changes. Recovery time calculations also indicated that BeC_19_@DMT and C_20_@DMT are better suited for filtration applications where rapid regeneration is not necessary. UV-Vis absorption spectroscopy showed that the maximum absorption wavelength for C_20_ and BeC_19_ shifts from the UV to the visible region after DMT adsorption, accompanied by a significant decrease in exciton energy. In contrast, MgC_19_ and CaC_19_ showed smaller spectral changes, indicating weaker photoresponsiveness.

NCI, ELF, LOL, and QTAIM analyses consistently confirmed that all interactions remain within the closed-shell, noncovalent regime. For C_20_@DMT, broad green interlocking surfaces indicated dominant van der Waals interactions, whereas BeC_19_@DMT showed distinct blue attractive regions around the Be site, indicating stronger localized interactions. QTAIM analysis revealed that BeC_19_@DMT exhibits the highest electron density at the bond critical point (ρ = 0.0674 a.u.) among the doped systems, while MgC_19_@DMT and CaC_19_@DMT showed significantly lower values (ρ = 0.0338 and 0.0271 a.u., respectively), further confirming the superior interaction strength of BeC_19_. Statistical validation using mean absolute deviation (MAD), root-mean-square deviation (RMSD), and Pearson correlation coefficients (*R* > 0.93 for all key properties) confirmed excellent agreement among the three computational methods, demonstrating that the observed qualitative trends are robust and method-independent.

Finally, based on these findings, we predict that pristine fullerene C20 can serve as an effective adsorbent for DMT removal, while BeC_19_, with its electronic disorder, significant conductivity modulation (a fourfold increase), and absorption in the visible region, can play a dual role as a strong adsorbent and an electrochemical/colorimetric sensor. Conversely, MgC_19_ and CaC_19_ exhibit weak interactions and minimal electronic response, making them unsuitable for sensing and adsorption applications. While the computational results demonstrate promising sensing and adsorption capabilities, extending these findings to pharmaceutical or toxicological applications requires additional studies, including ADMET, toxicity, and biological interaction analyses. Future work may incorporate such evaluations to assess the suitability of these nanostructures in biomedical applications. As this is a purely theoretical investigation, all reported values should be interpreted as qualitative trends rather than absolute benchmarks, and experimental validation is strongly encouraged to confirm the predictive capabilities of the proposed sensor candidates. These computational findings provide a valuable theoretical foundation and a strategic guide for future experimental efforts aimed at developing portable, selective, and cost-effective DMT sensors for forensic and clinical applications.

## Supplementary Information

Below is the link to the electronic supplementary material.

Supplementary Material 1

## Data Availability

The datasets used and/or analysed during the current study available from the corresponding author on reasonable request.
