# Supplementary material for: In silico insights into the adsorption and detection of dimethyltryptamine on metal doped C20 fullerene
Source: Sci Rep. 2026 Jul 22;16:28596. doi: 10.1038/s41598-026-60826-x (PMC13575126; doi:10.1038/s41598-026-60826-x)
Supplement: Supplementary file 1 [file 41598_2026_60826_MOESM1_ESM.docx]

The bond length between each of the nanocage and the indole nitrogen of DMT is shown in Figure S1. According to the results, when DMT is attached to each of the nanocage through the indole nitrogen (N2), it experiences a longer bond length than when it is attached through N1.

Fig S1. Optimized geometries of the C_20_@DMT, BeC_19_@DMT, MgC_19_@DMT, and CaC_19_@DMT complexes (at the B97D/lanl2dz, B97D/6-311+G(d), and wB97XD/lanl2dz levels of theory) showing the orientation of the indole nitrogen (N_2_) toward the pristine/ doped C_20_ cage.

Figure S2 shows the DOS plots calculated using the wB97XD/lanl2dz method for the attachment of DMT (from the N1 atom) to each of the nanocages. The DOS plots clearly confirm the HOMO and LUMO orbital energies and the energy gap for each of the designed structures and show good qualitative agreement with the results obtained with two other computational methods (B97D/lanl2dz and B97D/6-311+G(d)).

Fig S2. DOS plot calculated using the wB97XD/lanl2dz method for each of the structures designed in this work.

The two figures present the TDOS, PDOS (s and p contributions), and OPDOS profiles for the pristine and DMT-adsorbed systems (from the N1 atom in DMT) obtained using two different computational approaches (B97D/6-311+G(d) and wB97XD/lanl2dz). In both cases, the TDOS closely follows the p-orbital contribution, indicating that the electronic structure near the Fermi level is dominated by p states, while the s-orbital contribution remains relatively small. This behavior confirms that the interaction between the fullerene cages and DMT is primarily governed by p-type orbitals, consistent with π-type electronic interactions. A comparison between the pristine systems and their corresponding complexes shows that adsorption of DMT leads to noticeable changes in the electronic structure, particularly for C20 and BeC19. In these systems, the TDOS profiles are clearly modified near the Fermi level, and the OPDOS curves exhibit more pronounced positive and negative features, indicating stronger orbital overlap and enhanced electronic coupling between the adsorbent and the analyte. In contrast, MgC19 and CaC19 show only minor variations in their DOS profiles upon adsorption, and the OPDOS signals remain relatively weak, suggesting limited orbital interaction and weaker adsorption. Despite differences in the absolute energy scales between the two figures, which arise from the use of different computational methods, the overall trends remain consistent. The figure S2, associated with a functional that includes long-range exact exchange, shows larger energy gaps and more separated electronic states.

Fig S3. TODS and OPDOS spectra calculated using the wB97XD/lanl2dz method for each of the designed structures.

Fig S4. TODS and OPDOS spectra calculated using the B97D/6-311+G(d) method for each of the designed structures.

In order to ensure the reproducibility of reported data in the future, the X, Y, and Z coordinates were reported in Table S1.

Table S1. X, Y, Z coordinates of the structures designed in this work.

C20

%nprocshared=4

\# opt b97d/6-311+g(d) scrf=(cpcm,solvent=water) geom=connectivity

Title Card Required

0 1

C	1.16435600	0.99352700	1.28534000

C	-0.00615400	0.41464700	1.90712100

C	0.74096900	1.94259300	0.29264800

C	-0.79359400	1.92339600	0.29238100

C	-1.19139700	0.96246000	1.28459900

C	1.16969600	1.38518500	-0.93810900

C	-0.01296100	1.01413200	-1.68770500

C	-1.20535400	1.35290200	-0.93804000

C	1.96964900	0.13796000	-0.69322400

C	1.19131200	-0.96247300	-1.28462200

C	0.00614200	-0.41473600	-1.90710600

C	1.97308800	-0.08550900	0.69472800

C	1.20542200	-1.35284900	0.93800500

C	0.79359200	-1.92339900	-0.29235800

C	0.01296300	-1.01405100	1.68775400

C	-1.16968800	-1.38524800	0.93812600

C	-0.74095400	-1.94259600	-0.29259000

C	-1.16436300	-0.99350100	-1.28534300

C	-1.97315500	0.08547100	-0.69477500

C	-1.96956900	-0.13791000	0.69316700

1 2 1.5 3 1.5 12 1.0

2 5 1.5 15 1.5

3 4 1.0 6 1.5

4 5 1.5 8 1.5

5 20 1.0

6 7 1.0 9 1.0

7 8 1.0 11 1.5

8 19 1.0

9 10 1.0 12 1.5

10 11 1.5 14 1.5

11 18 1.5

12 13 1.0

13 14 1.5 15 1.0

14 17 1.0

15 16 1.0

16 17 1.5 20 1.0

17 18 1.5

18 19 1.0

19 20 1.5

20

C20@DMT (1)

%nprocshared=4

\# opt b97d/6-311+g(d) scrf=(cpcm,solvent=water) geom=connectivity

Title Card Required

0 1

C	-1.31200000	-1.52772900	1.69208500

C	-1.05847700	-0.08079700	1.74696900

C	-0.51768400	-2.06098400	0.60488900

C	0.16756100	-0.89746100	-0.02383300

C	-0.12675600	0.28883900	0.69816300

C	-1.41859000	-2.60415800	-0.34403800

C	-1.27507900	-1.78331200	-1.59099900

C	-0.30815100	-0.73602300	-1.35458100

C	-2.77212900	-2.41868000	0.18324500

C	-3.41651200	-1.48144000	-0.71545200

C	-2.50813200	-1.11050600	-1.80042900

C	-2.71502900	-1.81714900	1.53181800

C	-3.28246900	-0.43949700	1.32954200

C	-3.76644500	-0.28047000	0.00116300

C	-2.28985100	0.59222000	1.54044500

C	-2.16774300	1.43404700	0.32399600

C	-3.02881900	0.85549700	-0.61620400

C	-2.23304200	0.32771100	-1.71145600

C	-0.83923600	0.63286600	-1.60729500

C	3.51042500	-2.74494700	-0.97114500

C	3.24138700	-2.54352600	0.38454300

C	3.01083300	-1.22429700	0.80579800

C	3.06114700	-0.11425300	-0.09481800

C	3.33739200	-0.34920700	-1.45852600

C	3.55722100	-1.66003000	-1.88422500

H	3.68592300	-3.75738900	-1.33576700

H	3.19857900	-3.37797800	1.08437400

H	3.37501600	0.47614400	-2.17031200

H	3.76858900	-1.85844500	-2.93537200

C	2.74381400	1.06227600	0.66383700

C	2.53076900	0.64597100	1.96651200

N	2.69950300	-0.72101500	2.05355300

H	2.57218300	-1.26796100	2.89372700

C	2.60679800	2.44617400	0.11082000

C	1.43332800	2.67631300	-0.85825200

N	0.01210100	2.76239600	-0.26586100

C	0.00948100	3.35668000	1.11298100

H	0.53013400	4.31725200	1.08126300

H	-1.03130400	3.49291800	1.41698500

H	0.51115100	2.66555400	1.79183600

C	-0.79218700	3.65747300	-1.18013700

H	-1.82065200	3.69033100	-0.81363800

H	-0.34627200	4.65515900	-1.16182800

H	-0.75473400	3.22949800	-2.18486600

H	2.26904600	1.22055900	2.84814100

H	2.59175200	3.19350300	0.91293000

H	3.49319000	2.68648200	-0.49748600

H	1.59451200	3.63707600	-1.35617500

H	1.37508200	1.89732800	-1.62455200

C	-0.71406300	1.36678100	-0.22926600

1 2 1.0 3 1.0 12 1.5

2 5 1.0 15 1.5

3 4 1.0 6 1.5

4 5 1.5 8 1.5

5 50 1.0

6 7 1.0 9 1.0

7 8 1.5 11 1.5

8 19 1.0

9 10 1.0 12 1.0

10 11 1.0 14 1.5

11 18 1.0

12 13 1.0

13 14 1.5 15 1.5

14 17 1.0

15 16 1.0

16 17 1.5 50 1.0

17 18 1.0

18 19 1.5

19 50 1.0

20 21 1.5 25 1.5 26 1.0

21 22 1.5 27 1.0

22 23 1.5 32 1.5

23 24 1.5 30 1.5

24 25 1.5 28 1.0

25 29 1.0

26

27

28

29

30 31 2.0 34 1.0

31 32 1.5 45 1.0

32 33 1.0

33

34 35 1.0 46 1.0 47 1.0

35 36 1.0 48 1.0 49 1.0

36 37 1.0 41 1.0

37 38 1.0 39 1.0 40 1.0

38

39

40

41 42 1.0 43 1.0 44 1.0

42

43

44

45

46

47

48

49

50

C20@DMT (2)

%nprocshared=4

\# opt b97d/6-311+g(d) scrf=(cpcm,solvent=water) geom=connectivity b97d

Title Card Required

0 1

C	3.70334800	1.42971700	-0.42714400

C	2.25200000	1.59797800	-0.36502300

C	3.97577400	0.44589200	-1.44309700

C	2.64773300	-0.03305200	-1.93726200

C	1.60551200	0.70940700	-1.32675600

C	4.58882400	-0.64675600	-0.76160800

C	3.69663700	-1.82653300	-0.87957300

C	2.48427500	-1.40786500	-1.56714400

C	4.76485200	-0.29847100	0.63863000

C	3.93211600	-1.24070900	1.37269300

C	3.28679800	-2.16496000	0.43318500

C	4.28069300	1.08062000	0.87126700

C	3.05082600	0.85838700	1.66835500

C	2.89346500	-0.51146800	2.03271700

C	1.84486300	1.25141500	0.94722200

C	0.93222400	0.10884500	0.86299200

C	1.57876100	-0.97418600	1.49059400

C	1.83945400	-1.96570800	0.48207900

C	1.25182100	-1.66535200	-0.81512400

C	-1.67038300	3.56043200	-0.49128400

C	-1.06583900	2.44797900	-1.11468500

C	-1.45096100	1.19908100	-0.64738300

C	-2.39495400	0.99202600	0.36912300

C	-2.97068200	2.10927600	0.99857400

C	-2.59639000	3.39075700	0.55284200

H	-1.40563000	4.56291400	-0.82121600

H	-0.33450800	2.57040800	-1.90922500

H	-3.68147700	1.99545500	1.81325100

H	-3.03226700	4.27157900	1.02113800

C	-2.57321200	-0.45376400	0.54263100

C	-1.72800900	-1.08166000	-0.29230800

N	-0.90799600	-0.10640200	-1.03271500

H	-0.92920100	-0.27037900	-2.04350900

C	-3.59025900	-1.08681600	1.44339300

C	-5.03935100	-0.74295500	1.01100200

N	-5.33428300	-1.05620500	-0.39112300

C	-5.30313200	-2.50451400	-0.61215600

H	-6.01420900	-3.03785100	0.05639600

H	-5.56994900	-2.72849100	-1.65346000

H	-4.29641700	-2.89553300	-0.42348600

C	-6.65977300	-0.53381000	-0.74495300

H	-6.86885700	-0.75095300	-1.80081400

H	-7.46686400	-0.98293100	-0.12520000

H	-6.68184200	0.55422000	-0.59738700

H	-1.49313500	-2.12441900	-0.45396400

H	-3.43932400	-2.17397400	1.47605500

H	-3.47767300	-0.70464000	2.46817100

H	-5.73509400	-1.27464800	1.69652100

H	-5.19711700	0.33382500	1.15016500

C	0.65288800	-0.24429000	-0.60622600

1 2 1.0 3 1.5 12 1.0

2 5 1.0 15 1.5

3 4 1.0 6 1.5

4 5 1.5 8 1.5

5 50 1.0

6 7 1.0 9 1.0

7 8 1.0 11 1.5

8 19 1.0

9 10 1.0 12 1.0

10 11 1.0 14 1.5

11 18 1.0

12 13 1.0

13 14 1.5 15 1.0

14 17 1.0

15 16 1.0

16 17 1.5 50 1.0

17 18 1.5

18 19 1.0

19 50 1.0

20 21 1.5 25 1.5 26 1.0

21 22 1.5 27 1.0

22 23 1.5 32 1.0

23 24 1.5 30 1.0

24 25 1.5 28 1.0

25 29 1.0

26

27

28

29

30 31 2.0 34 1.0

31 32 1.0 45 1.0

32 33 1.0

33

34 35 1.0 46 1.0 47 1.0

35 36 1.0 48 1.0 49 1.0

36 37 1.0 41 1.0

37 38 1.0 39 1.0 40 1.0

38

39

40

41 42 1.0 43 1.0 44 1.0

42

43

44

45

46

47

48

49

50

BeC19

%nprocshared=4

\# opt b97d/6-311+g(d) scrf=(cpcm,solvent=water) geom=connectivity

Title Card Required

0 1

C	-0.68034900	1.54548300	-1.08375500

C	0.70441100	1.20699800	-1.43536600

C	-0.67943400	1.86270000	0.30636900

C	0.70614900	1.71040300	0.76818900

C	1.61904000	1.44851200	-0.33191800

C	-1.52973600	0.90280400	0.97194300

C	-0.68013800	0.16715200	1.88046700

C	0.70552300	0.63861400	1.76267800

C	-2.14810800	0.00199500	0.00109900

C	-1.53150500	-1.28979800	0.29584800

C	-0.68148200	-1.19564100	1.46013600

C	-1.53111000	0.39165100	-1.26522000

C	-0.68298500	-0.66544500	-1.76522900

C	-0.68363500	-1.71103100	-0.79558400

C	0.70271900	-0.19019600	-1.86678400

C	1.61521600	-1.01296100	-1.09046000

C	0.70185700	-1.84772200	-0.32868100

C	0.70314000	-1.52234700	1.09697300

C	1.61750100	-0.43945500	1.41965300

Be	2.62938900	-0.00257300	-0.00053800

1 2 1.0 3 1.5 12 1.5

2 5 1.0 15 1.0

3 4 1.0 6 1.5

4 5 1.0 8 1.0

5

6 7 1.5 9 1.0

7 8 1.0 11 1.5

8 19 1.0

9 10 1.0 12 1.0

10 11 1.5 14 1.5

11 18 1.0

12 13 1.5

13 14 1.5 15 1.0

14 17 1.0

15 16 1.0

16 17 1.0

17 18 1.0

18 19 1.0

19

20

BeC19@DMT (1)

%nprocshared=4

\# opt b97d/6-311+g(d) scrf=(cpcm,solvent=water) geom=connectivity out=wfn

Title Card Required

0 1

C	-1.78925900	-1.57387800	-1.28951600

C	-0.50454300	-0.92858200	-0.97297900

C	-2.63785600	-0.55057700	-1.80649000

C	-1.84611500	0.68792900	-1.78719000

C	-0.45835700	0.45508500	-1.41332600

C	-3.76721000	-0.42780900	-0.90955200

C	-3.71020400	0.90958100	-0.36201400

C	-2.52025800	1.60866900	-0.87591300

C	-3.70210000	-1.43347500	0.15303500

C	-3.46206400	-0.62698100	1.35370900

C	-3.52038600	0.78453000	1.04853100

C	-2.40166500	-2.07250000	-0.07856100

C	-1.48053400	-1.77362300	0.99451300

C	-2.14052200	-0.87643100	1.88608100

C	-0.30984400	-1.05244700	0.46603200

C	-0.12271600	0.23931400	1.11047900

C	-1.35357300	0.36597000	1.87531300

C	-2.22296100	1.41300700	1.34769800

C	-1.63869000	2.06796100	0.18823700

C	1.91886200	-3.55792400	-1.01297900

C	1.99067000	-3.29494200	0.35827800

C	2.42513300	-2.01556600	0.74955800

C	2.79180100	-1.00459300	-0.19802900

C	2.72078500	-1.30668800	-1.57477800

C	2.27991200	-2.57407800	-1.96982700

H	1.57532100	-4.53663700	-1.35339300

H	1.70761600	-4.04539800	1.09845100

H	2.99065000	-0.55762800	-2.32148900

H	2.21100800	-2.81375500	-3.03288400

C	3.14080700	0.17442500	0.55067900

C	2.98410200	-0.15113400	1.88551200

N	2.57510600	-1.46846700	2.00713000

H	2.32680100	-1.92271700	2.87711700

C	3.63087500	1.48367900	0.00161600

C	2.66672800	2.26648700	-0.91234200

N	1.39149200	2.77922800	-0.28613900

C	1.65187800	3.45102600	1.01976700

H	2.41330700	4.24110900	0.90550700

H	0.70897000	3.89137700	1.36912000

H	1.99040400	2.70272800	1.74511900

C	0.81470400	3.78307200	-1.23726200

H	-0.13069000	4.15404000	-0.82435100

H	1.52308100	4.61592700	-1.38416700

H	0.62243400	3.28445000	-2.19648400

H	3.15255100	0.45497200	2.77108900

H	3.96642000	2.13224500	0.82460400

H	4.52114800	1.31050700	-0.62932600

H	3.22086200	3.13885800	-1.30094000

H	2.35256400	1.64761200	-1.76351700

Be	0.12924300	1.57748500	-0.09027000

1 2 1.0 3 1.5 12 1.5

2 5 1.0 15 1.0

3 4 1.0 6 1.5

4 5 1.0 8 1.0

5 50 1.0

6 7 1.5 9 1.0

7 8 1.0 11 1.5

8 19 1.0

9 10 1.0 12 1.0

10 11 1.5 14 1.5

11 18 1.0

12 13 1.5

13 14 1.5 15 1.0

14 17 1.0

15 16 1.0

16 17 1.0 50 1.0

17 18 1.0

18 19 1.0

19 50 1.0

20 21 1.5 25 1.5 26 1.0

21 22 1.5 27 1.0

22 23 1.5 32 1.5

23 24 1.5 30 1.5

24 25 1.5 28 1.0

25 29 1.0

26

27

28

29

30 31 2.0 34 1.0

31 32 1.0 45 1.0

32 33 1.0

33

34 35 1.0 46 1.0 47 1.0

35 36 1.0 48 1.0 49 1.0

36 37 1.0 41 1.0 50 1.0

37 38 1.0 39 1.0 40 1.0

38

39

40

41 42 1.0 43 1.0 44 1.0

42

43

44

45

46

47

48

49

50

BeC19@DMT (2)

%nprocshared=4

\# opt b97d/6-311+g(d) scrf=(cpcm,solvent=water) geom=connectivity out=wfn

Title Card Required

0 1

C	3.92973000	1.51921800	-0.22145600

C	2.46465000	1.65026800	-0.21648300

C	4.25007800	0.61828700	-1.28022600

C	2.97007400	0.22455400	-1.88868500

C	1.84530200	0.96829300	-1.34296500

C	4.88971000	-0.53529200	-0.68697900

C	4.02819900	-1.66396800	-0.96210400

C	2.82984600	-1.21568700	-1.68790800

C	5.06890100	-0.35177700	0.75528000

C	4.15764200	-1.34610100	1.32775800

C	3.57208100	-2.16851700	0.29212900

C	4.37597600	0.91291400	1.01267900

C	3.19119900	0.70122400	1.81334900

C	3.05512300	-0.70570900	2.00945400

C	1.99846800	1.13391300	1.06874900

C	1.02861300	0.06149100	0.91039000

C	1.78309200	-1.09025700	1.37842700

C	2.10930800	-2.01308000	0.29357900

C	1.59903700	-1.55901200	-0.99168100

C	-1.97982200	3.58338900	-0.33557900

C	-1.38869800	2.55717700	-1.09281600

C	-1.73957100	1.24970700	-0.76131800

C	-2.63763200	0.92493400	0.27904400

C	-3.19980400	1.96406500	1.04463000

C	-2.86661900	3.28826900	0.72286300

H	-1.74047200	4.62274600	-0.56494400

H	-0.68122600	2.76978300	-1.89395700

H	-3.87886500	1.74686300	1.86938000

H	-3.29871300	4.10644000	1.30150600

C	-2.77127600	-0.53000400	0.31560200

C	-1.97775900	-1.03468000	-0.66505800

N	-1.22667100	0.01962800	-1.32199900

H	-1.25994600	-0.02236000	-2.34661500

C	-3.63998200	-1.31472500	1.25744000

C	-5.14201400	-0.97247700	1.11753200

N	-5.63633800	-1.05135900	-0.26197800

C	-5.55790700	-2.41621000	-0.78876800

H	-6.12573800	-3.14706600	-0.16695400

H	-5.97330600	-2.43423800	-1.80835200

H	-4.50935000	-2.74365200	-0.83928600

C	-7.01714000	-0.56545600	-0.32250500

H	-7.36856700	-0.58927600	-1.36621200

H	-7.71644600	-1.17750400	0.29355700

H	-7.05756500	0.47357200	0.04170400

H	-1.78979900	-2.06162800	-0.96118200

H	-3.47660200	-2.39032100	1.09705300

H	-3.35235200	-1.09985500	2.30063800

H	-5.71511900	-1.64434600	1.79842300

H	-5.30869200	0.05869000	1.46493200

Be	0.48024500	-0.12355800	-0.81452400

1 2 1.0 3 1.5 12 1.5

2 5 1.0 15 1.0

3 4 1.0 6 1.5

4 5 1.0 8 1.0

5 50 1.0

6 7 1.5 9 1.0

7 8 1.0 11 1.5

8 19 1.0

9 10 1.0 12 1.0

10 11 1.5 14 1.5

11 18 1.0

12 13 1.5

13 14 1.5 15 1.0

14 17 1.0

15 16 1.0

16 17 1.0 50 1.0

17 18 1.0

18 19 1.0

19 50 1.0

20 21 1.5 25 1.5 26 1.0

21 22 1.5 27 1.0

22 23 1.5 32 1.0

23 24 1.5 30 1.0

24 25 1.5 28 1.0

25 29 1.0

26

27

28

29

30 31 2.0 34 1.0

31 32 1.0 45 1.0

32 33 1.0 50 1.0

33

34 35 1.0 46 1.0 47 1.0

35 36 1.0 48 1.0 49 1.0

36 37 1.0 41 1.0

37 38 1.0 39 1.0 40 1.0

38

39

40

41 42 1.0 43 1.0 44 1.0

42

43

44

45

46

47

48

49

50

MgC19

%nprocshared=4

\# opt b97d/6-311+g(d) scrf=(cpcm,solvent=water) geom=connectivity

Title Card Required

0 1

C	0.91265500	-1.00361000	1.58491200

C	-0.48240000	-1.36656200	1.27350100

C	0.91240100	0.39542600	1.83313000

C	-0.48251900	0.84351200	1.66622800

C	-1.40931600	-0.27431300	1.54077400

C	1.77001300	1.01366000	0.85171100

C	0.91101400	1.87505300	0.07603100

C	-0.48364000	1.78617300	0.54733700

C	2.39771000	0.00006100	-0.00203800

C	1.76848200	0.23223100	-1.30629600

C	0.90954100	1.39066600	-1.25980800

C	1.76976100	-1.24533100	0.44973300

C	0.91076400	-1.78641200	-0.57526400

C	0.90928300	-0.87098900	-1.66211400

C	-0.48352700	-1.86477200	-0.10200400

C	-1.41195300	-1.19768600	-1.00570800

C	-0.48589200	-0.41921900	-1.81806700

C	-0.48597800	1.02116700	-1.56203500

C	-1.41257600	1.47261600	-0.53105300

Mg	-3.01691200	-0.00083500	0.00051500

1 2 1.0 3 1.5 12 1.5

2 5 1.0 15 1.0

3 4 1.0 6 1.5

4 5 1.0 8 1.0

5 20 1.0

6 7 1.5 9 1.0

7 8 1.0 11 1.5

8 19 1.0

9 10 1.0 12 1.0

10 11 1.5 14 1.5

11 18 1.0

12 13 1.5

13 14 1.5 15 1.0

14 17 1.0

15 16 1.0

16 17 1.0 20 1.0

17 18 1.0

18 19 1.0

19 20 1.0

20

MgC19@DMT (1)

%nprocshared=4

\# opt b97d/6-311+g(d) scrf=(cpcm,solvent=water) geom=connectivity out=wfn

Title Card Required

0 1

C	4.47552034	1.47919211	-0.21864302

C	3.03233923	1.65790113	0.00043700

C	4.60567635	0.65669105	-1.36412910

C	3.23838325	0.35476803	-1.81388714

C	2.24443817	1.14423209	-1.10703108

C	5.28206140	-0.54570004	-0.95945007

C	4.34619433	-1.61469612	-1.17986409

C	3.07331223	-1.08966809	-1.69669713

C	5.67565243	-0.47652404	0.44714803

C	4.81436637	-1.48026711	1.07055608

C	4.05585731	-2.19404917	0.07942201

C	5.07283439	0.78097906	0.88716407

C	4.00565230	0.54074304	1.82026014

C	3.84538729	-0.86167607	1.93394915

C	2.73321621	1.06113908	1.29758410

C	1.69337313	0.04606900	1.28127310

C	2.47943419	-1.16023809	1.47774511

C	2.61352420	-2.00757115	0.29782402

C	1.94060415	-1.51354612	-0.89148907

C	-2.69405621	3.81421529	-0.31426102

C	-2.04682516	2.78715821	-0.99880108

C	-2.37309218	1.48344211	-0.64596205

C	-3.31720125	1.17754109	0.35110403

C	-3.93852230	2.22402317	1.04482808

C	-3.62144528	3.53434927	0.70101306

H	-2.47142519	4.84425437	-0.56647404

H	-1.31876710	2.99855023	-1.77297614

H	-4.65642736	2.02173915	1.83102014

H	-4.09883831	4.35501233	1.22392910

C	-3.42941426	-0.27524102	0.42256403

C	-2.57956720	-0.77969506	-0.50098804

N	-1.82260014	0.26000602	-1.13432709

H	-1.74952813	0.18513501	-2.14791617

C	-4.32488833	-1.04912908	1.34497711

C	-5.81748643	-1.00159008	0.96057607

N	-6.11884647	-1.63593112	-0.32292102

C	-6.04668347	-3.09266924	-0.25389702

H	-6.78030253	-3.52024427	0.45495303

H	-6.24293049	-3.51621127	-1.24162610

H	-5.05147439	-3.41767226	0.05346100

C	-7.42080057	-1.21126509	-0.82953506

H	-7.59588559	-1.65503213	-1.81278014

H	-8.25802465	-1.50764012	-0.17047801

H	-7.44243556	-0.12439501	-0.93793607

H	-2.37953818	-1.80601014	-0.76767406

H	-3.98343130	-2.08523716	1.39987511

H	-4.23492832	-0.64563705	2.35956118

H	-6.40257348	-1.45665511	1.78144114

H	-6.13039444	0.04365000	0.89546907

Mg	0.38999703	0.04869600	-0.52032104

1 2 1.0 3 1.5 12 1.5

2 5 1.0 15 1.0

3 4 1.0 6 1.5

4 5 1.0 8 1.0

5 50 1.0

6 7 1.5 9 1.0

7 8 1.0 11 1.5

8 19 1.0

9 10 1.0 12 1.0

10 11 1.5 14 1.5

11 18 1.0

12 13 1.5

13 14 1.5 15 1.0

14 17 1.0

15 16 1.0

16 17 1.0 50 1.0

17 18 1.0

18 19 1.0

19 50 1.0

20 21 1.5 25 1.5 26 1.0

21 22 1.5 27 1.0

22 23 1.5 32 1.0

23 24 1.5 30 1.0

24 25 1.5 28 1.0

25 29 1.0

26

27

28

29

30 31 2.0 34 1.0

31 32 1.0 45 1.0

32 33 1.0 50 1.0

33

34 35 1.0 46 1.0 47 1.0

35 36 1.0 48 1.0 49 1.0

36 37 1.0 41 1.0

37 38 1.0 39 1.0 40 1.0

38

39

40

41 42 1.0 43 1.0 44 1.0

42

43

44

45

46

47

48

49

50

MgC19@DMT (2)

%nprocshared=4

\# opt b97d/6-311+g(d) scrf=(cpcm,solvent=water) geom=connectivity out=wfn

Title Card Required

0 1

C	-2.42349200	-2.07644100	-0.00753000

C	-1.13660000	-1.52475500	0.45469700

C	-2.60276600	-1.60086600	-1.33699300

C	-1.41816700	-0.77354900	-1.63790300

C	-0.37953100	-0.89581400	-0.62304800

C	-3.80322500	-0.79961800	-1.35274300

C	-3.37280200	0.53715500	-1.69507100

C	-1.90845500	0.58834900	-1.86252800

C	-4.46488700	-0.81735700	-0.04245300

C	-4.28572800	0.55894700	0.42910200

C	-3.67290500	1.37984600	-0.58903900

C	-3.51639100	-1.56929300	0.78858000

C	-2.90678700	-0.71290600	1.77768300

C	-3.38757400	0.60789800	1.55924700

C	-1.44395500	-0.65525700	1.59519900

C	-0.95134200	0.71462800	1.48517500

C	-2.20135000	1.42686400	1.24730500

C	-2.38175400	1.91776200	-0.12082200

C	-1.28360500	1.61748600	-1.03527300

C	2.85213500	-3.54878700	-1.11015600

C	2.14092400	-3.31680500	0.07266000

C	2.32752300	-2.07547400	0.70230200

C	3.18945700	-1.06666200	0.16923400

C	3.90876100	-1.33576800	-1.01671700

C	3.73135500	-2.57239200	-1.64580200

H	2.73072200	-4.50161700	-1.62871600

H	1.46500900	-4.06557600	0.48841100

H	4.59034700	-0.59285400	-1.43487800

H	4.27824900	-2.79339000	-2.56440200

C	3.09860800	0.07975400	1.05051700

C	2.21011800	-0.27637000	2.05725400

N	1.77582200	-1.57423600	1.86698800

H	0.97596400	-1.97678000	2.34186400

C	3.87299000	1.36628400	0.93690800

C	3.52902600	2.17073400	-0.33160900

N	2.10568100	2.61294100	-0.39084800

C	1.84498200	3.67309900	0.61935600

H	2.52036100	4.53410400	0.46139900

H	0.80202400	4.00531100	0.52094600

H	1.99478100	3.27199000	1.62975100

C	1.83251900	3.16836700	-1.74409200

H	0.77882700	3.47143800	-1.79501300

H	2.48649900	4.03613500	-1.94874500

H	2.01766700	2.38691400	-2.49490900

H	1.87903700	0.29562800	2.91772500

H	3.71737400	1.98013900	1.83624200

H	4.95435400	1.15264300	0.88749100

H	4.18770400	3.05599300	-0.40838800

H	3.70307200	1.54048400	-1.21514900

Mg	0.64708200	0.98284400	-0.05727000

1 2 1.0 3 1.5 12 1.5

2 5 1.0 15 1.0

3 4 1.0 6 1.5

4 5 1.0 8 1.0

5 50 1.0

6 7 1.5 9 1.0

7 8 1.0 11 1.5

8 19 1.0

9 10 1.0 12 1.0

10 11 1.5 14 1.5

11 18 1.0

12 13 1.5

13 14 1.5 15 1.0

14 17 1.0

15 16 1.0

16 17 1.0 50 1.0

17 18 1.0

18 19 1.0

19 50 1.0

20 21 1.5 25 1.5 26 1.0

21 22 1.5 27 1.0

22 23 1.5 32 1.0

23 24 1.5 30 1.0

24 25 1.5 28 1.0

25 29 1.0

26

27

28

29

30 31 1.5 34 1.0

31 32 1.5 45 1.0

32 33 1.0

33

34 35 1.0 46 1.0 47 1.0

35 36 1.0 48 1.0 49 1.0

36 37 1.0 41 1.0

37 38 1.0 39 1.0 40 1.0

38

39

40

41 42 1.0 43 1.0 44 1.0

42

43

44

45

46

47

48

49

50

CaC19

%nprocshared=4

\# opt b97d/6-311+g(d) scrf=(cpcm,solvent=water) geom=connectivity

Title Card Required

0 1

C	-1.16135400	1.63743300	-0.91195800

C	0.23692900	1.34441800	-1.28850700

C	-1.16096100	1.80650600	0.49828400

C	0.23689100	1.61103400	0.93362800

C	1.17351300	1.55057200	-0.18583200

C	-2.02217400	0.79628400	1.06415500

C	-1.15910400	-0.02953200	1.87464000

C	0.23836800	0.44480000	1.80753300

C	-2.64630200	-0.00143600	0.00043300

C	-2.02121300	-1.32122900	0.15906500

C	-1.15852100	-1.33565400	1.31647500

C	-2.02243500	0.52140300	-1.22212400

C	-1.15972100	-0.47271500	-1.81398900

C	-1.15903300	-1.60933300	-0.96225900

C	0.23763800	0.00427800	-1.86176900

C	1.17469900	-0.93389800	-1.24816900

C	0.23876300	-1.78694300	-0.51935000

C	0.23943100	-1.61326800	0.92758900

C	1.17579300	-0.61166200	1.43250000

Ca	3.21563800	-0.00031800	-0.00010300

1 2 1.0 3 1.5 12 1.5

2 5 1.0 15 1.0

3 4 1.0 6 1.5

4 5 1.0 8 1.0

5 20 1.0

6 7 1.5 9 1.0

7 8 1.0 11 1.5

8 19 1.0

9 10 1.0 12 1.0

10 11 1.5 14 1.5

11 18 1.0

12 13 1.5

13 14 1.5 15 1.0

14 17 1.0

15 16 1.0

16 17 1.0 20 1.0

17 18 1.0

18 19 1.0

19 20 1.0

20

CaC19@DMT (1)

%nprocshared=4

\# opt b97d/6-311+g(d) scrf=(cpcm,solvent=water) geom=connectivity out=wfn

Title Card Required

0 1

C	3.36113900	-1.20774300	-1.54045400

C	2.20678500	-0.33922200	-1.82894100

C	2.99619100	-1.98959700	-0.41834500

C	1.62991600	-1.57528900	-0.05556400

C	1.02563800	-0.70166300	-1.05460500

C	3.91803600	-1.68830900	0.64381100

C	3.12360500	-1.09939100	1.68806300

C	1.71086700	-1.01071600	1.28028800

C	4.94493000	-0.75103700	0.18561100

C	4.63623500	0.46814400	0.93469400

C	3.56699200	0.23290000	1.86739000

C	4.50863700	-0.42262900	-1.17257400

C	4.07909000	0.94761200	-1.24993500

C	4.15758600	1.49767800	0.05199800

C	2.66211900	1.02781200	-1.64515800

C	1.86603200	1.82023200	-0.71554000

C	2.78645500	1.89744600	0.41267400

C	2.41168400	1.09495100	1.56433400

C	1.17442100	0.33855500	1.40991700

C	-2.68800400	-3.81527500	1.00219500

C	-2.48003500	-3.49635600	-0.32925500

C	-2.91528900	-2.24660800	-0.76827100

C	-3.54127800	-1.31296300	0.09750800

C	-3.74397800	-1.67001700	1.43958900

C	-3.31788200	-2.91200200	1.87811400

H	-2.36302000	-4.77964700	1.37537300

H	-2.00049400	-4.19363400	-1.00641300

H	-4.23222500	-0.98809100	2.12651100

H	-3.47181900	-3.19740500	2.91249200

C	-3.85511800	-0.14614700	-0.69005800

C	-3.43200600	-0.42200300	-1.95982600

N	-2.86517900	-1.67370900	-2.01248300

H	-2.46864500	-2.09416700	-2.83693000

C	-4.60611100	1.07609200	-0.24646600

C	-3.91657100	1.97135600	0.79427900

N	-2.69481500	2.68142100	0.34576200

C	-2.98075700	3.58159000	-0.78743500

H	-3.78625700	4.29227900	-0.54802500

H	-2.08154200	4.15001800	-1.03226900

H	-3.26915400	3.00960800	-1.66914800

C	-2.19885700	3.48469600	1.48148800

H	-1.29955700	4.03078400	1.18963700

H	-2.94932700	4.21497000	1.81872100

H	-1.95389700	2.82991900	2.32018200

H	-3.50627900	0.18080900	-2.85172600

H	-4.89151900	1.66610700	-1.12104400

H	-5.55126100	0.76600700	0.21542100

H	-4.65374100	2.71387100	1.14061000

H	-3.63770000	1.37300300	1.66505500

Ca	-0.57532200	1.15895100	-0.28242800

1 2 1.0 3 1.5 12 1.5

2 5 1.0 15 1.0

3 4 1.0 6 1.5

4 5 1.0 8 1.0

5 50 1.0

6 7 1.5 9 1.0

7 8 1.0 11 1.5

8 19 1.0

9 10 1.0 12 1.0

10 11 1.5 14 1.5

11 18 1.0

12 13 1.5

13 14 1.5 15 1.0

14 17 1.0

15 16 1.0

16 17 1.0 50 1.0

17 18 1.0

18 19 1.0

19 50 1.0

20 21 2.0 25 1.5 26 1.0

21 22 1.5 27 1.0

22 23 1.5 32 1.5

23 24 1.5 30 1.5

24 25 2.0 28 1.0

25 29 1.0

26

27

28

29

30 31 2.0 34 1.0

31 32 1.5 45 1.0

32 33 1.0

33

34 35 1.0 46 1.0 47 1.0

35 36 1.0 48 1.0 49 1.0

36 37 1.0 41 1.0

37 38 1.0 39 1.0 40 1.0

38

39

40

41 42 1.0 43 1.0 44 1.0

42

43

44

45

46

47

48

49

50

CaC19@DMT (2)

%nprocshared=4

\# opt b97d/6-311+g(d) scrf=(cpcm,solvent=water) geom=connectivity out=wfn

Title Card Required

0 1

C	5.06132900	1.51760400	-0.32664000

C	3.64833400	1.65844800	0.06448200

C	5.07266700	0.69127100	-1.47593300

C	3.66629000	0.35230800	-1.75251200

C	2.73487000	1.12295900	-0.93751100

C	5.82763100	-0.49080300	-1.15769100

C	4.89525000	-1.58154400	-1.25429300

C	3.55374000	-1.08949700	-1.61170900

C	6.38033500	-0.40200600	0.19476100

C	5.63163300	-1.42679200	0.92412600

C	4.77403200	-2.15965500	0.03215400

C	5.80919000	0.84647500	0.70227300

C	4.86553800	0.58190200	1.75469600

C	4.75578100	-0.82272700	1.89177100

C	3.52406700	1.06498300	1.38487200

C	2.50582700	0.02800000	1.49840300

C	3.35067900	-1.15538000	1.60148000

C	3.36218000	-2.00374100	0.42178800

C	2.52731400	-1.53646800	-0.67821700

C	-2.97818200	3.57830900	-0.23985600

C	-2.67541700	2.57196300	-1.14818200

C	-3.17462600	1.29179100	-0.88731100

C	-3.96706400	1.00454800	0.26035200

C	-4.25190600	2.04585000	1.16162600

C	-3.75756000	3.31748100	0.90558400

H	-2.60787900	4.58218000	-0.41490400

H	-2.07481900	2.77200900	-2.02850100

H	-4.84880600	1.86031300	2.04816300

H	-3.97292100	4.12642500	1.59480400

C	-4.29450700	-0.40050500	0.21552100

C	-3.70392800	-0.89252100	-0.92106300

N	-3.03128400	0.11496300	-1.58681500

H	-2.51957500	0.00056000	-2.44689400

C	-5.12834900	-1.16313200	1.20431200

C	-6.64518900	-0.90612200	1.09639800

N	-7.23975900	-1.35593300	-0.16331900

C	-7.34508600	-2.80955500	-0.24044200

H	-7.99663900	-3.23043700	0.54867400

H	-7.75930500	-3.09664400	-1.20996400

H	-6.36206800	-3.27424300	-0.14947100

C	-8.54251600	-0.73630400	-0.38504300

H	-8.93858800	-1.04661700	-1.35544900

H	-9.28751400	-1.00780500	0.38649200

H	-8.44212400	0.35164300	-0.39082100

H	-3.70901600	-1.89637100	-1.31713900

H	-4.92462400	-2.23303600	1.10775800

H	-4.82953600	-0.89327400	2.22401100

H	-7.14685900	-1.37719000	1.96347200

H	-6.82097600	0.16986200	1.17899200

Ca	0.56222100	0.01483000	-0.16387300

1 2 1.0 3 1.5 12 1.5

2 5 1.0 15 1.0

3 4 1.0 6 1.5

4 5 1.0 8 1.0

5 50 1.0

6 7 1.5 9 1.0

7 8 1.0 11 1.5

8 19 1.0

9 10 1.0 12 1.0

10 11 1.5 14 1.5

11 18 1.0

12 13 1.5

13 14 1.5 15 1.0

14 17 1.0

15 16 1.0

16 17 1.0 50 1.0

17 18 1.0

18 19 1.0

19 50 1.0

20 21 1.5 25 1.5 26 1.0

21 22 1.5 27 1.0

22 23 1.5 32 1.5

23 24 1.5 30 1.5

24 25 1.5 28 1.0

25 29 1.0

26

27

28

29

30 31 2.0 34 1.0

31 32 1.0 45 1.0

32 33 1.0

33

34 35 1.0 46 1.0 47 1.0

35 36 1.0 48 1.0 49 1.0

36 37 1.0 41 1.0

37 38 1.0 39 1.0 40 1.0

38

39

40

41 42 1.0 43 1.0 44 1.0

42

43

44

45

46

47

48

49

50

The adsorption energies for the formation of the R-C_20_@DMT complex (R=Be, Mg, Ca) in which DMT is bonded to each of the pristine structures (C_20_, BeC_19_, CaC_19_, and MgC_19_) through the indole nitrogen (N_2_) are reported in Table S2. As the results show, when DMT is bonded to the R-C_20_ structures through the N_2_, the adsorption energy of the complex formation is significantly lower than when DMT is bonded to the R-C_20_ structures through the N_1_.

| Table S2. Calculated adsorption energies due to the binding of each of the C_20_, BeC_19_, CaC_19_ and MgC_19_ structures to DMT (which are oriented towards the indole nitrogen (N_2_) in DMT). All values are in kcal.mol^-1^. | | | | | | |
| --- | --- | --- | --- | --- | --- | --- |
| Structure | **B97D/lanl2dz** | | **B97D/6-311+G(d)** | | **WB97XD/lanl2dz** | |
|  | Eads | E_BSSE_ | Eads | E_BSSE_ | Eads | E_BSSE_ |
| C_20_@DMT | -16.66 | 5.11 | -12.08 | 1.84 | -19.90 | 7.81 |
| BeC_19_@DMT | -20.49 | 4.50 | -22.95 | 2.43 | -9.86 | 16.77 |
| CaC_19_@DMT | -3.32 | 1.30 | -9.58 | 1.19 | -2.75 | 0.68 |
| MgC_19_@DMT | -6.46 | 1.87 | -9.51 | 1.51 | -5.29 | 1.75 |
